# Diverse cytomotive actins and tubulins share a polymerization switch mechanism conferring robust dynamics

**DOI:** 10.1126/sciadv.adf3021

**Published:** 2023-03-29

**Authors:** James Mark Wagstaff, Vicente José Planelles-Herrero, Grigory Sharov, Aisha Alnami, Frank Kozielski, Emmanuel Derivery, Jan Löwe

**Affiliations:** ^1^ MRC Laboratory of Molecular Biology, Francis Crick Avenue, Cambridge CB2 0QH, UK.; ^2^ Department of Pharmaceutical and Biological Chemistry, School of Pharmacy, University College London, 29-39 Brunswick Square, London WC1N 1AX, UK.

## Abstract

Protein filaments are used in myriads of ways to organize other molecules within cells. Some filament-forming proteins couple the hydrolysis of nucleotides to their polymerization cycle, thus powering the movement of other molecules. These filaments are termed cytomotive. Only members of the actin and tubulin protein superfamilies are known to form cytomotive filaments. We examined the basis of cytomotivity via structural studies of the polymerization cycles of actin and tubulin homologs from across the tree of life. We analyzed published data and performed structural experiments designed to disentangle functional components of these complex filament systems. Our analysis demonstrates the existence of shared subunit polymerization switches among both cytomotive actins and tubulins, i.e., the conformation of subunits switches upon assembly into filaments. These cytomotive switches can explain filament robustness, by enabling the coupling of kinetic and structural polarities required for cytomotive behaviors and by ensuring that single cytomotive filaments do not fall apart.

## Teaser

Actin- and tubulin-like protein structures reveal shared conformation switches upon polymerisation, enabling cytomotive dynamics.

## INTRODUCTION

**Fig. 1.**
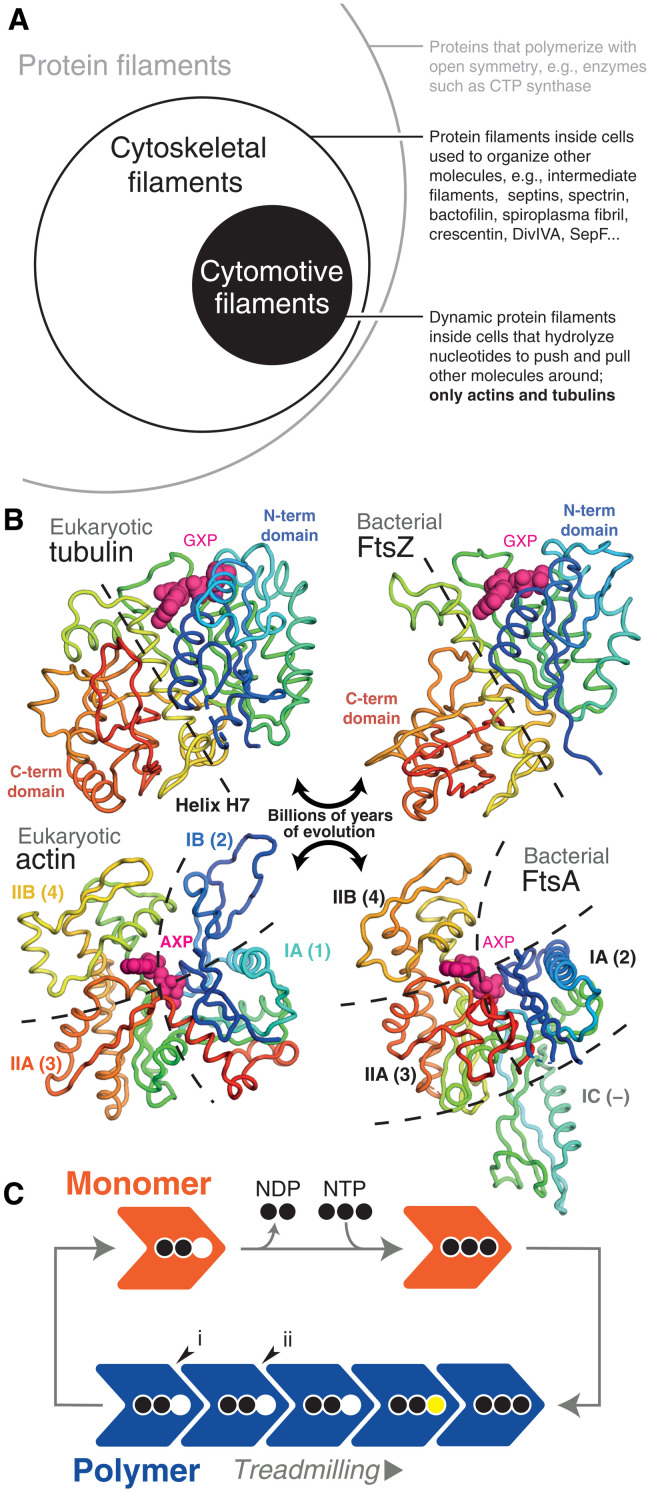
Actin and tubulin superfamilies form cytomotive filaments. (**A**) Cytomotive filaments are a subset of cytoskeletal filaments, themselves a subset of all protein filaments. Only actin and tubulin superfamily proteins are known to form intrinsically (i.e., not requiring accessory factors for complex dynamics) cytomotive filaments (circles not to scale). (**B**) Despite billions of years of evolution separating subfamily members in both cases, members of both actin and tubulin superfamilies retain highly conserved structural cores. In the case of tubulins, N- and C-terminal domains are linked by a conserved helix, H7 (yellow). In the case of actins, the core comprises subdomains IA, IIA, and IIB (also referred to as 1, 3, and 4). Structures clockwise from top left are PDB IDs 5NQU, 3VOA, 4A2A, and 5JLF. (**C**) Dynamic properties of cytomotive filaments, such as treadmilling, arise due to coupling of nucleotide hydrolysis and polymerization cycles. One property of the model shown is that the same bonds can be formed/broken upon addition/loss of subunits at either end such that structural and kinetic polarities are not intrinsically coupled (see also fig. S12). In addition, severing at interfaces (i, end) and (ii, middle) should be expected to proceed at the same rate.

Protein filaments are used widely in fundamental cell biological processes across the tree of life. Through polymerization, nanometer-sized protein subunits form larger structures—filaments—used to organize other molecules at a wide range of scales, up to that of eukaryotic cells many micrometers across (and sometimes much larger). Polymerization is therefore one mechanism by which microscopically encoded information in the genome is able to manipulate the macroscopic world. Many protein filaments function through dynamic cycles of assembly and disassembly, characteristically using energy released through inbuilt nucleotide hydrolysis activity to perform work by pushing and pulling other molecules around. These dynamic filaments have been termed cytomotive, to distinguish them among the wider class of cytoskeletal filaments that perform organizing functions (itself a subset of all filament-forming proteins) (Fig. 1A) (*1*). So far, we know of only two protein families that form cytomotive filaments and have inbuilt nucleotide hydrolysis activity—the actin and tubulin superfamilies (Fig. 1B). These superfamilies encompass the diverse array of homologs of eukaryotic actin and tubulin, which are found in almost all bacterial and archaeal cells, performing a wide variety of roles in cellular processes (*2*).

Our understanding of how actin and tubulin filaments are able to perform useful work revolves around two dynamic filament behaviors, treadmilling and dynamic instability. Basic models for both behaviors envision a nucleotide-state switch: Nucleotide triphosphate (NTP)—bound subunits polymerize at growing filament ends, hydrolysis occurs while subunits are within filaments, and nucleotide diphosphate (NDP)–bound subunits leave from shrinking filament ends—because NDP interfaces are thermodynamically less favorable (Fig. 1C) (*3*–*5*). The reality is clearly more complicated—these behaviors turn out to be complex and many details remain to be understood.

At a fundamental level, the filaments described by this model exhibit two key problems. First, when considering a single-stranded protofilament, loss of an NDP-bound end subunit is thermodynamically identical to breakage of an NDP interface within the filament, and so, depolymerization and breakage are expected to occur at the same rate: In a regime where depolymerization is functionally relevant, the filament will often fall apart. Second, and less straightforwardly, the simple nucleotide-state switch model does not permit for reliable coupling of structural and kinetic polarities (*6*). In other words, while a filament of this type can grow and shrink (Fig. 1C), the way in which it does so will be determined stochastically, by early hydrolysis events. Both problems would appear to severely limit the usefulness of a filament within a cell—and motivate efforts to understand the mechanistic basis of dynamic filament behaviors via a range of methods.

Both problems are reduced if a multistranded filament is formed, comprising multiple protofilaments bound together by lateral interactions (*5*, *7*). Severing of multiple protofilaments becomes thermodynamically different to losing a single end subunit, and simultaneous formation of different combinations of lateral and longitudinal interactions would appear to offer a way to robustly couple structural and kinetic polarities. Not all cytomotive actins and tubulins are multistranded, however (*2*).

Modeling of eukaryotic actin and microtubule (MT) filament dynamics across a range of scales has attracted much attention and has generated insights into many cytoskeleton-associated processes (*3*, *4*, *8*–*10*). Hitherto, such modeling and experimental work have largely been restricted to the well-conserved eukaryotic actin and tubulin proteins and the multistranded filaments they form. We posit that this approach has limited progress in understanding the basis of cytomotivity.

Recent years have shown us that actin and tubulin superfamily members from bacteria and archaea also exhibit cytomotive functionality and that the filaments they form encompass a great deal of structural diversity (*2*). To improve our understanding of the basis of cytomotivity, present as it is across the tree of life, we have undertaken a comparative structural biology approach: both assembling and analyzing existing data and collecting previously unknown structural data.

## RESULTS

### Cytomotive actins share a conformation switch upon polymerization

**Fig. 2.**
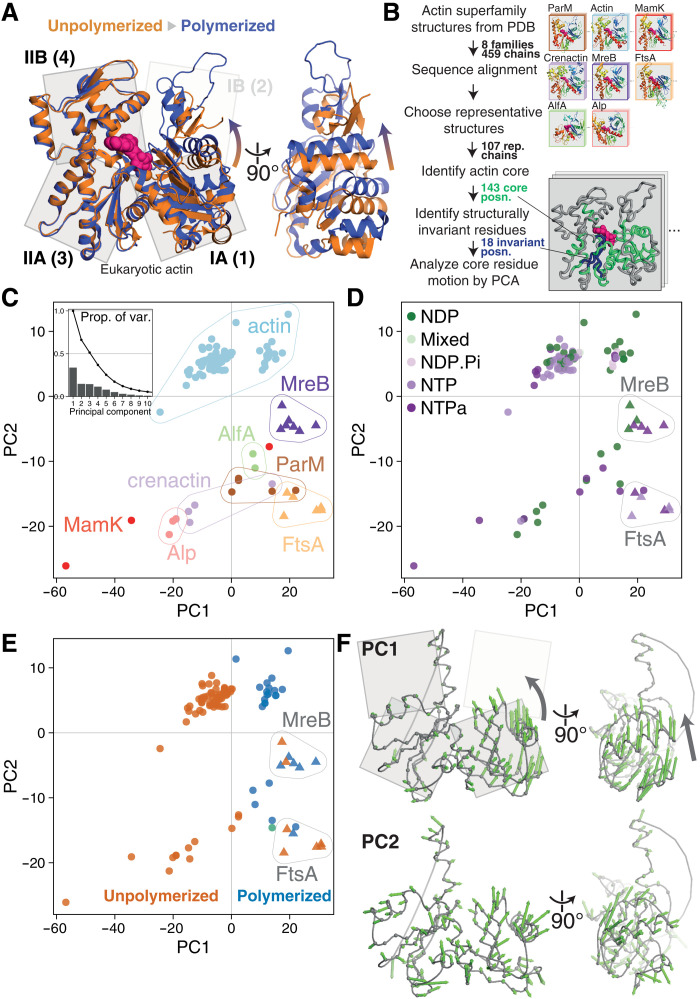
Conformational analysis of actin superfamily structures reveals a conserved subunit switch upon polymerization. (**A**) Inspection of eukaryotic actin subunit structures in unpolymerized (PDB 3EL2, orange) and polymerized (actin-tropomyosin, 5JLF, blue) states reveals the previously characterized actin “propeller twist” conformational change. (**B**) Pipeline for PCA of conformational changes across the actin superfamily. Bottom right inset: Example structure with identified actin core positions (green) and structurally invariant core (blue). (**C**) Results of PCA. Representative structures [colored by subfamily, triangles mark FtsA/MreB structures in (C) to (E)] are plotted in PC1-PC2 subspace. PC2 mostly describes the differences between subfamilies, with further contributions from PC3 (fig. S1). Inset: Proportion of variance explained by each component. (**D**) Identical to (C), but structures are colored by the hydrolysis state of the bound nucleotide (NTPa, less/nonhydrolyzable nucleotide triphosphate analog). (**E**) Identical to (C) and (D), but structures are colored by polymerization state (unpolymerized in orange, polymerized in blue; green: PDB 4A62, ParM:ParR, discussed in text S1). PC1 mostly describes the polymerization state of subunits, with exception of the MreB and FtsA subfamilies (triangles), which form noncytomotive filaments. (**F**) Per-position PC loading vectors (green arrows) for PC1 and PC2 are visualized on a representative actin core [see scheme in (B) and Materials and Methods]. Both views as in (A).

Structures have been determined for a number of actin superfamily members, apparently representing complete sets of snapshots for their assembly/polymerization cycles. This includes eukaryotic actin, archaeal crenactin, and bacterial proteins MamK, ParM, FtsA, and MreB. All of these proteins share a core of structurally conserved subdomains (Figs. 1B and 2A). The core polymerizes in various ways to form diverse filament architectures (*2*, *11*).

To our knowledge, this wealth of structural information has not previously been analyzed systematically. To do so, we performed a structure-guided sequence alignment and structural superposition of all Protein Data Bank (PDB)–deposited actin superfamily structures, before selecting representative structures and analyzing conformational flexibility at conserved amino acid positions using principal components analysis (PCA) (Fig. 2B). PCA reduces complex datasets to a small number of maximally descriptive dimensions. PCA can be a powerful tool for the analysis of protein conformations, as high-dimensional variation in the position of equivalent atoms in related structures can often be compressed to informative trajectories representing inter- and intradomain motions (*12*). More details can be found in Materials and Methods.

The first three, most descriptive, principal components (PC1, PC2, and PC3) of the actin superfamily dataset explain ~65% of the variance in amino acid α carbon (Cα) positions (Fig. 2C, inset, and fig. S2). PC2 and PC3 largely describe the differences between actin subfamilies (Fig. 2C and fig. S1; interesting outliers are discussed in text S1). On the other hand, PC1 describes differences within subfamilies.

If common structural mechanisms underpin the functionality of actin superfamily members, we expect equivalent functional states of different family members to be colocated within PC subspaces. Nucleotide hydrolysis states are often ascribed as defining functional states in protein conformational cycles. Examination of actin PC1-PC2 subspaces does not reveal notable evidence of a correlation between backbone conformation and nucleotide hydrolysis state (Fig. 2D). In contrast, subunit polymerization state is clearly associated with the value of PC1 (Fig. 2E). Two almost nonoverlapping clusters of structures are seen along PC1: These are polymerized actin and actin-like subunits, and monomeric ones, i.e., the structures of assembled actin superfamily subunits are systematically, and discretely, different from those of monomeric subunits. We argue that gross structural variation within the superfamily relating to function can be summarized as performance of a conformational subunit switch upon polymerization, described by PC1.

The subunit switch in eukaryotic actin is well characterized as the “propeller twist” or “monomer flattening” of subdomains IA/B (also referred to as 1/2) versus IIA/B (3/4) upon polymerization [reviewed in (*13*, *14*)]. The notable finding from our analysis is that PC1, the most descriptive component, succinctly describes the conformational switch upon polymerization across the entire actin superfamily, despite the billions of years of evolution separating the subfamilies, and the substantial variation in subdomain composition and longitudinal filament architecture within them. Our analysis also generalizes the result that nucleotide hydrolysis state does not define subunit conformation, previously demonstrated for eukaryotic actin (*15*, *16*).

We can visualize PCs as trajectories through structure space, as they describe correlated linear displacements of amino acids (Fig. 2F and movie S1). The conserved switch can be seen in the PC1 trajectory as a closing up of the two halves of the monomer, by hinging between subdomains IA/B. The PC2 trajectory is less concerted, mostly describing differences in secondary structure packing between families.

Bacterial proteins MreB and FtsA (structures are plotted as triangles in Fig. 2, C to E) are an important pair of exceptions to this overall pattern. For these proteins, both monomeric and polymerized subunits are placed in the region of PC1-PC2 subspace where we otherwise find only polymerized subunits from the other families. These protein families therefore appear to have a “jammed” subunit switch. Crucially, these proteins are thought to form static, scaffolding filaments—i.e., they are noncytomotive (*17*, *18*). Therefore, we note that the subunit switch, performed upon polymerization, is restricted to cytomotive filament-forming actin superfamily members.

### Solution structures of *Drosophila* tubulin heterodimers confirm the presence of a subunit switch and the absence of a nucleotide-driven switch

**Fig. 3.**
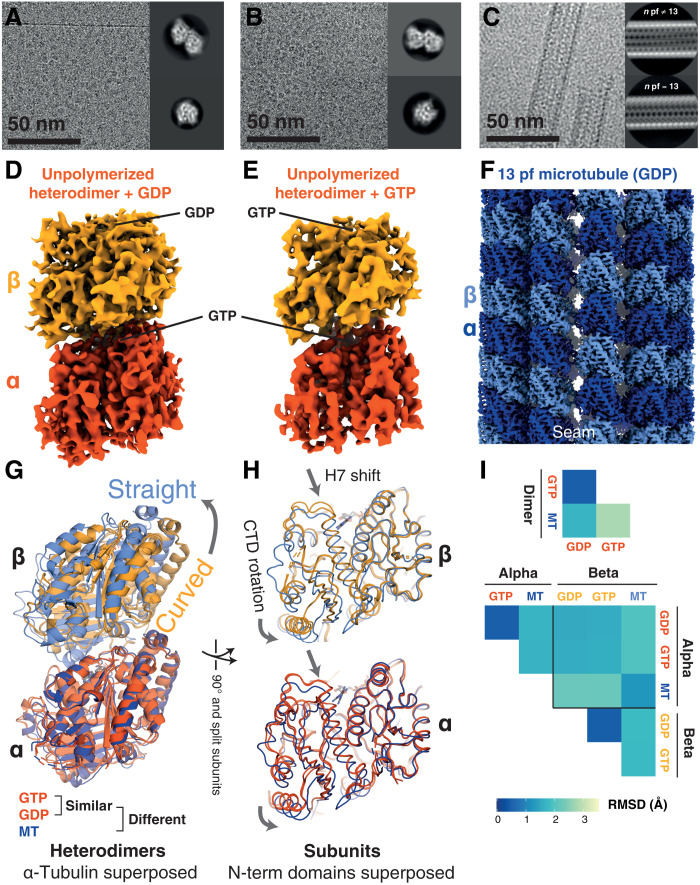
Cryo-EM structures of free/un-complexed *D. melanogaster* (*Dm*) tubulin heterodimers and an MT recapitulate classical tubulin assembly switches and reiterate the absence of a nucleotide state-driven switch. (**A**) Cryo-EM study of *Dm* tubulin heterodimers prepared with GDP. Representative micrograph and 2D classes. Processing scheme can be found in fig. S3. (**B**) Cryo-EM study of *Dm* tubulin heterodimers prepared with GTP. Representative micrograph and 2D classes. Processing scheme can be found in fig. S4. (**C**) Cryo-EM study of *Dm* MTs prepared with GTP. Representative micrograph and 2D classes. (**D** and **E**) Cryo-EM maps of *Dm* tubulin prepared with GDP or GTP occupying the β-tubulin binding site as indicated. (**F**) Cryo-EM map of *Dm* tubulin polymerized into 13 protofilament MT. (**G** and **H**) Comparison of *Dm* models (PDB; GTP: 7QUD, GDP: 7QUC, and MT: 7QUP) from the dimer maps (orange: dark—α subunit, light—β subunit) and the 13-protofilament MT (blue shades). Structures in (G) are aligned on the N-terminal domain of α-tubulin; structures in (H) are aligned on the N-terminal domains of the respective subunits. (**I**) Cα RMSD comparison of dimers (top) and individual subunit structures (bottom), following superposition as in (G) and (H). Unpolymerized heterodimers are very similar to one another.

Our observations of the actin superfamily led us to analyze the tubulin superfamily, the other class of proteins known to form cytomotive filaments. Despite decades of work, uncertainty remains about the overall functional-conformational landscape of eukaryotic tubulin heterodimers, particularly as to the role of nucleotide hydrolysis and to the implications of the sparse sampling of crystal forms resolved for unpolymerized heterodimers (*19*, *20*). Here, using cryo–electron microscopy (cryo-EM), we solved solution-state structures of recombinant *Drosophila melanogaster* tubulin heterodimers, purified in both guanosine diphosphate (GDP)– and guanosine triphosphate (GTP)–bound states, in addition to a structure of MTs polymerized from the same material (Fig. 3, A to F).

Structures of GDP- and GTP-bound heterodimers (α-tubulin: nonexchangeable GTP, β-tubulin GDP/GTP) were highly similar to each other (Fig. 3, G to I) and to published crystal structures of other eukaryotic tubulin heterodimers [e.g., all-atom root mean square deviation (RMSD) between our GDP-occupied structure by cryo-EM and a designed ankyrin repeat protein-bound heterodimer by x-ray crystallography (PDB 5EYP) is 1.0 Å], confirming that nucleotide hydrolysis state does not play a direct role in determining gross heterodimer conformation (*21*). Comparison of solution-state heterodimer structures to structures of heterodimers within MTs recapitulated results seen for other eukaryotic tubulins: As a whole, the heterodimer transitions from a curved to straight conformation upon polymerization (Fig. 3G). Meanwhile, within both monomers, N- and C-terminal domains rotate relative to one another in both monomers, in each case accompanied by a downward shift of helix 7 (H7) (Fig. 3H). The curved-to-straight heterodimer transition and the within-monomer between-domains rotation have each been described as a “tubulin assembly switch” (*21*–*24*).

The complex properties of MTs, both in their dynamics and architecture, being formed of heterodimers and comprising many protofilaments, present a formidable barrier to inferring the mechanistic principles underpinning those dynamic behaviors. We sought to disentangle mechanisms underlying the idiosyncratic properties of MTs from the more general mechanisms and behaviors of tubulin superfamily protofilaments by examining simpler systems.

### Structures of tubulin superfamily protofilaments support generality of subunit switch

While eukaryotic MTs are complex in structure and composition, and arose in evolution relatively recently—close in time to eukaryogenesis—the tubulin fold is ancient and can act in isolation as a molecular motor (*25*). One-subunit-thick FtsZ protofilaments, formed of identical monomers, have been observed to treadmill in vitro and in vivo (*26*–*28*)—and therefore appear to be minimal cytomotive filaments, and a good place to look for clues as to the mechanistic principles underpinning tubulin superfamily cytomotivity.

Many crystal structures of FtsZ have been solved, all of which correspond to monomeric, unassembled FtsZ subunits, except for a few crystal forms of *Staphylococcus aureus* (*Sa*) FtsZ (*29*), in which SaFtsZ crystallizes in straight protofilaments extending through the crystals. The “open” conformation of the subunits within the *S. aureus* filaments is markedly different to the “closed” conformation of the monomeric crystal forms and to those from other species. The monomeric closed form is related to the polymeric open form by a C-terminal domain (CTD) rotation and a shift of H7—similar to the relationship between corresponding eukaryotic tubulin monomers within curved and straight heterodimers as described in the previous section.

We and others previously crystallized monomeric *S. aureus* FtsZ in a closed conformation, and we produced a low-resolution cryo-EM structure of *E. coli* FtsZ filaments, which showed that subunits within the filament also adopt the open form seen in *S. aureus* FtsZ crystals (*6*, *30*). Despite extensive efforts, we have been unable to produce a high-resolution cryo-EM structure of frozen-hydrated FtsZ filaments. Here, we present a second intermediate resolution structure of a filament from a different organism, *Mycobacterium tuberculosis* (fig. S5). This map confirms that polymerized subunits are in the distinctive open conformation. Together, these data demonstrate that single-stranded bacterial FtsZs, much like eukaryotic tubulins, undergo a conformational switch upon polymerization—an analogy suggested previously (*22*) and further supported by the work of others (*30*, *31*).

While the conformational switch upon polymerization seen in single-stranded FtsZ suggests that multi-strandedness of eukaryotic tubulin MTs may not be a critical component of the mechanism underlying filament cytomotivity, it does not rule it out.

Straight single protofilaments of eukaryotic tubulin have been observed under certain conditions, but their structures have not been resolved at high resolution, e.g., by Elie-Caille *et al.* (*32*). Similarly, we were unable to generate single-protofilament (1pf) eukaryotic tubulin samples for structural determination.

**Fig. 4.**
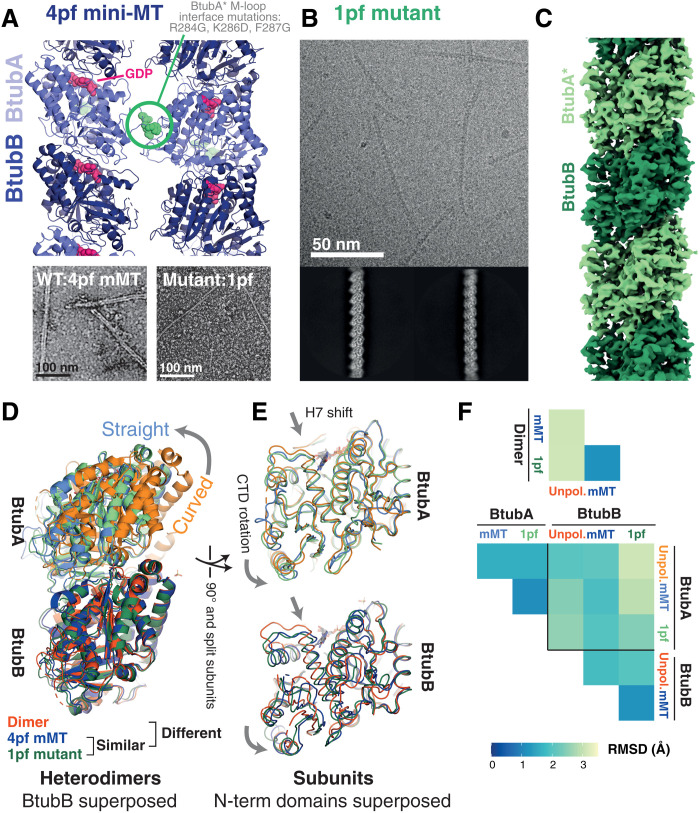
Cryo-EM structures of a single protofilament tubulin reveals a polymerization-associated conformational switch. (**A**) M-loop mutations in BtubA prevent lateral interactions between protofilaments (pf). Top: Side view of four-stranded mini-MT structure determined previously by cryo-EM (PDB 5O0C). M-loop residues 284 to 286 are shown as spheres in green. Bottom: Micrographs of negatively stained specimens: Left: 4pf mini-MT formed by WT BtubAB. Right: Single protofilament/1pf polymers formed by BtubA*B M-loop mutant (BtubA*: R284G, K286D, and F287G). (**B**) Cryo-EM study of BtubA*B polymerized using GMPCPP. Representative micrograph and 2D class averages are shown. Processing scheme can be found in fig. S7. Single filaments are also formed with GTP (see fig. S8). (**C**) Cryo-EM map of a single protofilament formed of BtubA*B polymerized using GMPCPP. (**D** and **E**) Comparison of BtubAB models from the dimeric (unpolymerized) crystal form (orange, PDB 2BTQ), the 4pf wt mini-MT (mMT, blue, PDB 5O09), and the single protofilament (1pf, M-loop mutant, PDB 7QUQ) solved here (green). Structures in (D) are aligned on the N-terminal domains of BtubB; structures in (E) are aligned on the N-terminal domains of the respective subunits. (**F**) Cα RMSD comparison of dimers (top) and individual subunit structures (bottom), following superposition as in (D) and (E). Polymerized heterodimers are highly similar.

We thus used a different system of reduced complexity: bacterial tubulin A/B (BtubAB). *Prosthecobacter dejongeii* BtubAB heterodimers form four-stranded (4pf) mini-MTs, which exhibit dynamic instability and treadmilling (*33*). We designed a mutant of BtubAB, intended to decouple longitudinal polymerization from lateral assembly interactions by introducing multiple mutations into the M-loop because lateral interactions are exclusively via the “MT” M-loop of BtubA. We termed this mutant BtubA*B (Fig. 4A; BtubA*: R284G, K286D, and F287G). BtubA*B polymerizes in the presence of GTP and GMPCPP to form straight single protofilaments (1pf) (Fig. 4, A to C, and fig. S8).

Using cryo-EM, we resolved a 3.5-Å reconstruction of single protofilaments of BtubA*B protein polymerized with GMPCPP (Fig. 4, B and C). Comparison of the 1pf model built into the reconstructed density with the previously determined 4pf mini-MT wild-type (WT) structure (Fig. 4, D to F) revealed that the 1pf and 4pf polymer structures were essentially identical. Comparison of the polymerized structures and the published crystal structure of WT unpolymerized heterodimers (PDB 2BTQ) as for *Dm* tubulin illustrated the previously characterized curved to straight transition of the dimer upon polymerization and the within-subunit interdomain rotation and H7 shift.

Our explorations of simpler-than-MT tubulin systems therefore appeared to confirm the straightforward conclusion that the conformation changes seen within eukaryotic tubulin heterodimers upon polymerization are not determined by the multistranded nature of MTs. Using three different systems also strengthened the case that the within-subunit conformation changes are shared between diverse tubulin superfamily members. To generalize further, we proceeded to perform a superfamily structural analysis analogous to that completed for the actins above.

### Subunit switching upon polymerization is a property of cytomotive tubulins

Tubulin superfamily members are variously used in a wide range of critical cellular processes across the tree of life (*2*). Structural studies have been used to interrogate mechanistic details underlying many of these functions, yielding a wealth of data describing the structural plasticity of this ancient fold. As for the actin superfamily, to our knowledge, the tubulin superfamily structural dataset has not previously been systematically analyzed in its totality.

**Fig. 5.**
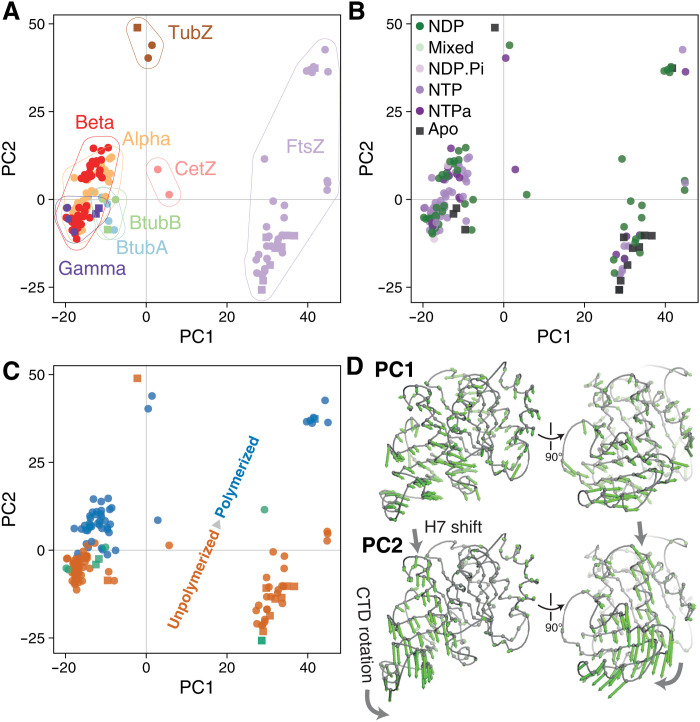
Conformational analysis of tubulin superfamily structures reveals a conserved subunit switch upon assembly. (**A**) Results of PCA. Representative tubulin structures plotted in PC1-PC2 subspace, colored by tubulin subfamily. PC1 mostly describes differences between subfamilies. (**B**) As (A), but structures/points are colored by nucleotide hydrolysis state. (**C**) As (A) and (B), but structures/points are colored by assembly state [unpolymerized, orange; polymerized, blue; “special/ambiguous,” green (more details in text S1)]. PC2 mostly describes the assembly state. (**D**) Per-position PC loading vectors for PC1 and PC2 (green arrows) are visualized on a representative tubulin core (see Materials and Methods).

All deposited tubulin superfamily structures were therefore analyzed in this work, alongside the structures determined here. The methodology described above for the actins was followed: Representative structures were selected, and residues were placed into a common frame of reference via a structure-guided sequence alignment, before determination of a common, invariant structural core and subsequent analysis of conformational differences via PCA (Fig. 5 and fig. S10).

As for the actins, the PCA is successful in compressing the structural variation seen across the superfamily, with PC1 and PC2 alone describing 80% of the variance in Cα positions among the representative structures. Examining the PC1-PC2 subspace, it is clear that for the tubulins, PC1 mostly describes differences between subfamilies (Fig. 5A), while PC2 describes differences within subfamilies corresponding to conformational changes upon polymerization (Fig. 5B). Again, as for the actins, the hydrolysis state of bound nucleotides does not appear to correlate with position in the PC subspace (Fig. 5C)—and is therefore not obviously a determinant of gross conformation.

Visualization of the trajectories corresponding to PC1 and PC2 on representative structures (Fig. 5D and movie S2) shows that PC1 describes the wholesale “widening” of the monomer fold seen between the eukaryotic tubulins and FtsZs, while PC2 describes the within-monomer interdomain rotation and accompanying shift in H7, observed upon polymerization of *Dm* tubulin, *Mtb* FtsZ, and BtubAB in the structural studies described above.

Unusual structural states can be quickly identified in the PC1-PC2 subspace, details of several of these are discussed in text S1 and can be inspected in fig. S11. Overall, however, the picture is clear: The tubulin superfamily, much like the actin superfamily, appears to have a shared subunit conformational switch upon polymerization. The role of conformational changes within the divergent viral TubZ family is less apparent from this analysis; our working hypothesis is that these proteins are (like MreB and FtsA) “jammed” switches, and that their cytomotive properties (*34*) are explained by an alternative mechanism driven by the unusual structural interactions between subunits along and possibly across protofilaments via long C-terminal tails.

## DISCUSSION

We set out to improve our understanding of the mechanisms underpinning cytomotive behaviors of protein filaments. Our approach was to complete a structural survey of the actin and tubulin superfamilies not only via the assembly of existing datasets but also by generating previously unknown data to fill important gaps. We hypothesized that such an approach would yield insights not only due to the unusual relevance of structural methods for examining polymerization as a protein function but also due to the richness of existing data—with many sets of structures comprising complete, or almost complete, snapshots of functional cycles for individual subfamilies being available.

Our dataset comprised actin and tubulin superfamily members from across the tree of life: including but extending beyond the well-studied eukaryotic actins and eukaryotic tubulins. This meant that the data incorporated a great deal of structural and functional diversity, allowing us to draw conclusions about the presence of general mechanisms underpinning cytomotive behavior seen in these superfamilies.

In summary, the approach taken suggested that subunit assembly switches exist within both actin and tubulin superfamilies, and in each case, the switching mechanism is to a great extent shared among the superfamily. In both superfamilies, these assembly switches have been identified previously within individual subfamilies—as too in many cases have analogies been drawn between subfamilies [for example, in the cases of FtsZ and eukaryotic tubulin (*22*), and ParM and eukaryotic actin (*35*)].

We conclude that in both superfamilies, the assembly switch is a crucial feature for understanding dynamic filament properties. We ultimately suggest that the cytomotive behaviors common to the two superfamilies may be shared because of the analogous assembly switches.

Previously, we set out the narrower claim that the treadmilling behavior of single-stranded polymers of the prokaryotic tubulin FtsZ can be explained by considering the properties of a model filament composed of subunits that perform a conformation switch upon assembly (*6*). This was subsequently supported by detailed agent-based modeling (*36*). Since then, similar arguments have been advanced for understanding the behavior of both eukaryotic actin and MTs (*37*, *38*).

On a simplistic level, we note that the two challenges for naïve models of assembly (outlined in Introduction) are not faced by models that incorporate a subunit assembly switch: The presence of the switch avoids both filament-splitting occurring at the same rate as end-subunit leaving and filament kinetic polarity being uncoupled from structural polarity such that the directions of filament growth and shrinkage are determined stochastically (fig. S12).

In other words, a subunit assembly switch provides a mechanism by which critical concentrations for assembly can be made reliably different at the two ends of filaments (*3*, *4*, *39*), thus making the filaments useful. We do not claim that a subunit switch is the only way to implement a useful cytomotive filament, but we do think it is striking that, despite large variations in overall filament architecture, the presence of subunit switches in both superfamilies overlaps closely with the presence of cytomotivity. There is still substantial work to be done in integrating findings regarding structural variation within filaments (*16*, *40*), and perhaps finding other implementations of cytomotive behavior (for instance, the possibility of along-filament interactions driving end differences in TubZ).

**Fig. 6.**
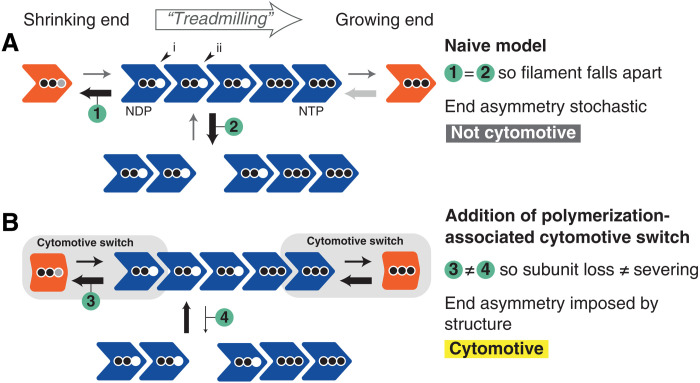
A polymerization-associated subunit switching mechanism, the cytomotive switch is required for robust single-stranded filament dynamics. Two models for protein filament polymerization are shown. In (**A**), subunits are rigid. In (**B**), subunits perform a cytomotive switch, i.e., they have two conformations: one compatible with being in a filament but that is unstable when unpolymerized (blue), and a second that is incompatible with polymerization but is stable when unpolymerized (orange). Arrows of the same appearance indicate rates that are identical; their widths are proportional to the rates they represent. Three black dots denote NTP-loaded subunits; two black dots mean NDP. A gray dot means exchange of NDP/NTP. The filament in (A) faces two problems, which render it not usefully cytomotive; the filament in (B) uses a cytomotive switch to solve both problems. End asymmetry/coupling of kinetic and structural polarities is explained further in fig. S12.

We also note that nucleotide binding and hydrolysis play a central role in the summary model shown in Fig. 6. NTP-bound subunits form stronger longitudinal bonds, while built-in hydrolysis to form NDP allows these interactions to be weakened, permitting far from equilibrium dynamics powered by the cell’s metabolism and providing the overall energetic driver.

Complete understanding of the mechanisms underlying the remarkable dynamics of cytomotive filaments remains out of reach—especially for more complex filament architectures. Much information is missing about thermodynamics, kinetics, and fine-grained structural dynamics at filament ends. However, we anticipate that recognizing the generality of the gross conformational subunit switch can lend clarity and support accurate interpretation of old and new data. Recent work elucidating the structure and dynamics of tubulin homologs from Odinarcheota (*41*), a deeply branching clade comprising the closest known prokaryotic relatives of eukaryotes, revealed that subunits of this presumed intermediate between ancient FtsZs and modern tubulins undergo a characteristic conformational switch upon polymerization.

Despite the remaining uncertainties and the work ahead, we think it is reasonable to suggest that actin and tubulin proteins have persisted over many billion years during evolution because their subunits are all-in-one implementations of both a polymerization-coupled conformation switch and built-in nucleotide hydrolysis, together allowing them to do useful work in cells that goes beyond forming a scaffolding “cytoskeleton.” We propose the name “cytomotive switch” to describe polymerization-associated conformation switches within nucleotide-hydrolyzing subunits of cytomotive filaments.

## MATERIALS AND METHODS

All reagents were purchased from Sigma-Aldrich unless otherwise specified.

### Protein expression and purification

The amino acid sequences of the proteins used are listed in text S2.

### Recombinant *D. melanogaster* αβ-tubulin heterodimers

A codon-optimized gene coding for tubulin α1 84B (GenBank entry NM_057424) carrying a tandem N-terminal His_6_-tag and a Protein C epitope tag (PC tag, EDQVDPRLIDGKG) was custom-synthesized (Twist Bioscience) and cloned into a pMT Puro vector (*42*). The resulting vector was used to generate a D.mel-2 stable cell line adapted to grow in suspension in serum-free Insect-Xpress medium (Lonza). Briefly, cells were transfected using Effectene (Qiagen) and selected in Insect-Xpress medium supplemented with puromycin (5 μg/ml; Gibco). After a couple of days of selection, transgene expression was induced by the addition of 0.6 mM CuSO_4_ to the medium. The cells were kept in Insect-Xpress medium supplemented with puromycin (5 μg/ml) and 0.6 mM CuSO_4_ for at least a week, keeping the cell density in the range of 5 × 10^6^ to 25 × 10^6^ cells/ml. Cells were collected by centrifugation; washed in 20 mM Hepes (pH 7.6), 150 mM KCl, and 1 mM CaCl_2_ buffer; frozen in liquid N_2_; and stored at −80°C. Note that using this procedure, only the α-tubulin is overexpressed, and this is enough to recover functional αβ-tubulin heterodimers, as the recombinant α-tubulin associates with endogenous β-tubulin.

To purify the αβ-tubulin heterodimers, the frozen cells were thawed and diluted in tubulin lysis buffer [80 mM K-Pipes, 10 mM CaCl_2_, 10 μM Na-GTP, 10 μM MgCl_2_, benzamidine (0.12 mg/ml), chymostatin (20 μg/ml), antipain (20 μg/ml), leupeptin (0.5 μg/ml), 0.24 mM Pefabloc SC, and 0.5 mM phenylmethylsulfonyl fluoride (pH 6.9)] and lysed by extrusion using a dounce homogenizer. Lysed cells were rocked for 1 hour at 4°C to ensure complete MT depolymerization and then clarified by centrifugation at 66,000*g* for 30 min using a JA 25.50 rotor (Beckman). The clarified lysate was incubated with 2 ml of preequilibrated Protein C affinity resin (Roche) for 3 hours at 4°C. The resin was then packed into an empty column (Bio-Rad) and washed with 50 ml of tubulin wash buffer [80 mM K-Pipes, 10 μM Na-GTP, 10 μM MgCl_2_, and 1 mM CaCl_2_ (pH 6.9)], 50 ml of tubulin adenosine triphosphate (ATP) buffer (wash buffer supplemented with 10 mM MgCl_2_ and 10 mM Na-ATP), 50 ml of low-salt buffer (wash buffer + 50 mM KCl), 50 ml of high-salt buffer (wash buffer + 300 mM KCl), 50 ml of Tween buffer (wash buffer + 0.1% Tween 20 and 10% glycerol), and finally 50 ml of tubulin wash buffer without CaCl_2_. Recombinant tubulin was eluted by incubating the resin with 2 ml of tubulin buffer [80 mM K-Pipes, 10 μM Na-GTP, 10 μM MgCl_2_, and 5 mM EGTA (pH 6.9)]. Multiple elution steps were performed, and the fractions were analyzed by SDS–polyacrylamide gel electrophoresis (SDS-PAGE). The protein-containing fractions were pooled and further purified on a Superdex 200 column (GE HealthCare), equilibrated, and eluted in tubulin buffer. The final pool was concentrated using AMICON Ultra-4 centrifugal units and used directly for cryo-EM sample preparation. Mass spectrometry analysis confirmed an isotopically pure composition of the copurified β-tubulin (tubulin β1-56D, UniProt TBB1_DROME, 19 unique peptides, 58% coverage). Similarly, tubulin α1-84B (UniProt TBA1_DROME) is the only expected isotype of α-tubulin in this preparation because it is the isotype we expressed as a fusion with the Protein C–affinity tag used in the first affinity step. The absence of endogenous, untagged α-tubulin was confirmed by mass spectrometry through the absence of α-tubulin peptides in the β-tubulin band analyzed above (untagged α-tubulins would migrate at the same molecular weight as the β1-56D tubulin, on the contrary to the Protein C–fused α1-84B tubulin, which migrates slower because of the tag.

### GDP exchange of *D. melanogaster* αβ-tubulin heterodimers

To exchange the nucleotide present in β-tubulin, concentrated pure αβ-tubulin heterodimers were incubated with 5 mM EDTA for 1 hour on ice. After the incubation, 20 mM Na-GDP and 20 mM MgCl_2_ were added to the sample. The exchanged tubulin was then injected onto a Superdex 200 3.2/300 column (GE HealthCare), equilibrated, and eluted in 80 mM K-Pipes, 100 μM Na-GDP, 100 μM MgCl_2_, and 5 mM EGTA (pH 6.9) to remove any residual GTP present in the sample. The protein was analyzed by SDS-PAGE, pooled, and concentrated for cryo-EM.

### *M. tuberculosis* FtsZ

For cryo-EM experiments, *M. tuberculosis* FtsZ was freshly expressed and purified as previously described, with small modifications (*43*). Briefly, full-length *ftsZ* (coding for residues 1 to 379), subcloned into expression vector pProEx, was expressed in BL21(DE3) pLysS, and cells were harvested by centrifugation. The supernatant was discarded, and cells were resuspended in buffer A [50 mM Hepes (pH 7.2), 300 mM NaCl, 5% glycerol, and 10 mM imidazole] and lysed on ice by sonication. The lysate was centrifuged at 20,000*g* for 15 min at 4°C.

The resulting supernatant was loaded into a 5-ml His-Trap FF column, preequilibrated with buffer A. The column was washed with 100 ml of buffer B [50 mM Hepes (pH 7.2), 150 mM NaCl, 5% glycerol, and 50 mM imidazole], and the protein was eluted with buffer C [50 mM Hepes (pH 7.2), 150 mM NaCl, 5% glycerol, and 250 mM imidazole].

The protein was dialyzed overnight at 4°C in the presence of PreScission protease (1 mg of PreScission protease for 50 mg of FtsZ), in cleavage buffer [50 mM Hepes (pH 7.2), 150 mM NaCl, 1 mM EDTA, 0.01% Tween 20, and 1 mM dithiothreitol (DTT)]. Cleaved FtsZ was further purified by applying a second HisTrap FF column and was found in the flow-through. FtsZ was then dialyzed in dialysis buffer [25 mM Hepes (pH 7.2), 0.1 mM EDTA, 10 mM DTT, 50 mM NaCl, and 5% glycerol], concentrated to 15.5 mg/ml, aliquoted, frozen in liquid nitrogen, and stored at −80°C.

### *P. dejongeii* BtubA*B

BtubAB proteins from *P. dejongeii* were coexpressed and copurified according to previously published protocols (*33*, *44*), with some modifications to improve purity given that smaller single protofilaments were to be imaged. The three mutations in the M-loop of the BtubA subunit (R284G, R286D, and F287G—the triple mutant is henceforth denoted BtubA*) were designed on the basis of the BtubAB mini MT cryo-EM structure [PDB 5O09; (*33*)] to stop interactions with subunits from neighboring protofilaments. They were introduced by mutagenic polymerase chain reaction (Q5 mutagenesis kit, New England Biolabs) of the BtubAB-expressing plasmid (*44*), using the primers CCGTTGACACCGCCAGACGGCAGTGATGGTGAGGAATTGGGCATTGAG and AGCAAAGGCGCACATGAGGAAGTGCAGCGA, and blunt ligation of the product.

Six liters of 2xTY medium containing ampicillin (100 μg/ml) was inoculated with transformed C41(DE3) *E. coli* cells from three overnight selective TY 90-mm plates. The cultures were grown until mid-log phase at 36°C and induced with 1 mM isopropyl-β-d-thiogalactoside for 3 hours at 36°C while shaking in 2-liter flasks at 190 rpm. The cells were harvested by centrifugation, frozen in liquid nitrogen, and stored at −80°C. The cells were thawed and resuspended in 300 ml of buffer A [20 mM tris-HCl and 1 mM sodium azide (pH 8.5)]. Six EDTA-free protease inhibitor tablets (Roche) and small amounts of solid deoxyribonuclease I (Sigma-Aldrich) were added. The cells were opened using a cell disruptor at 25 kPSI (Constant Systems). Cleared lysate was obtained by centrifugation in a Beckman 45 Ti rotor at 35,000 rpm for 30 min. The supernatant was applied to two 5-ml HiTrap Q XL (Cytiva) columns at 5 ml/min, which had been equilibrated in buffer A. Protein elution was achieved using stepwise increases of the concentration of buffer B (buffer A + 1 M NaCl). Most of BtubA*B were eluted at 25% buffer B as determined by SDS-PAGE of the resulting fractions. Fractions containing BtubA*B were concentrated using centrifugal concentrators with a 10-kDa molecular weight cutoff (Vivaspin 20, Sartorious) and applied to a Sephacryl S300 16/60 column (Cytiva), equilibrated in buffer C [20 mM tris and 1 mM EDTA (pH 7.5)]. Fractions were again checked by SDS-PAGE and concentrated as before. The sample was diluted 10-fold with buffer A (pH 8.5) and applied to a Mono Q 4.6/100 column (Cytiva) and equilibrated in buffer A. Proteins were eluted with a 40-column volume linear gradient to 100% buffer B, and most of BtubA*B was again eluted at around 25% buffer B but with increased purity. Fractions were checked by SDS-PAGE and concentrated as before using centrifugal concentrators to around 500 μl and 42 mg/ml, as determined by the ultraviolet absorption of the concentrated sample and a calculated molar extinction coefficient. Aliquots of the sample were flash-frozen in liquid nitrogen and stored at −80°C.

### Cryo-EM sample preparation and imaging

For all imaging, a nominal defocus range of −1.0 to −3.0 μm was used. All Quantifoil/UltrAuFoil grids were purchased from Quantifoil Micro Tools GmbH.

### GTP-bound *D. melanogaster* αβ-tubulin heterodimers

*D. melanogaster* tubulin heterodimers, purified in the presence of GTP as above, were diluted into cold BRB buffer [80 mM Pipes (pH 6.9), 1 mM MgCl_2_, and 1 mM EGTA] with GTP (50 μM) to a final concentration of 0.05 mg/ml. A total of 2.5 μl was applied to a gold-on-gold grid (UltrAuFoil R2/2 200 mesh), prepared with a single layer of graphene oxide (*45*). After a wait of 30 s, the grid was blotted on both sides for 4 s and then vitrified by plunge freezing using Vitrobot Mark IV (FEI) into liquid ethane maintained at 93.0 K using an ethane cryostat (*46*). The Vitrobot chamber temperature was set to 10°C, and humidity was set to 100%. Micrograph movies were collected using a Titan Krios transmission electron microscope (TEM) (FEI) operating at 300 kV, with a K3 detector (Gatan Inc.). Pixel size was 0.86 Å, dose per frame was adjusted to 1 e^−/^Å^2^, and 40 frames were recorded. A total of 4042 movies were collected over two sessions.

### GDP-bound *D. melanogaster* αβ-tubulin heterodimers

*D. melanogaster* tubulin heterodimers, purified in the presence of GTP and then exchanged into buffer containing GDP as above, were diluted into cold BRB buffer [80 mM Pipes (pH 6.9), 1 mM MgCl_2_, and 1 mM EGTA] also with GDP (50 μM) to a final concentration of 0.05 mg/ml. Grids were prepared as for the GTP-bound sample. Micrograph movies were collected using a Titan Krios TEM (FEI) operating at 300 kV, with a K3 detector (Gatan Inc.). Pixel size was 0.86 Å, dose per frame was adjusted to 1 e^−^/Å^2^, and 40 frames were recorded. A total of 5724 movies were collected in one session.

### *D. melanogaster* MTs

To prepare dynamic *D. melanogaster* MTs, 5 μM of purified αβ-tubulin heterodimers was diluted in polymerization buffer [80 mM K-Pipes, 10% dimethyl sulfoxide, 1 mM GTP, and 1 mM MgCl_2_ (pH 6.9)], supplemented with 10 μM Taxol to favor MT nucleation. Five microliters of this reaction was then added to a 35-μl solution of 25 μM αβ-tubulin heterodimers in polymerization buffer (without Taxol) and incubated at 37°C for 20 min to induce MT polymerization. Note that the final Taxol concentration in this sample is ca. 1 μM. After the incubation, the protein was layered on top of a cushion solution [80 mM K-Pipes, 1 mM GTP, 1 mM MgCl_2_, and 60% glycerol (pH 6.9)] and centrifuged at 100,000*g* for 30 min at 37°C using a warm TLA100 fixed-angle rotor (Beckman). After the spin, the top solution was removed and the interface with the cushion solution was washed with EM buffer [80 mM K-Pipes, 1 mM GTP, and 1 mM MgCl_2_ (pH 6.9)]. The cushion solution was then removed, and the MT pellet was washed with 3 × 100 μl warm EM buffer to remove any residual glycerol from the solution. The pellet was then resuspended in warm EM buffer, and 3 μl of this sample was applied to a carbon-on-gold grid (Quantifoil R2/2 Au 200 mesh), recently glow-discharged for 1 min at 30 mA. After a wait of 30 s, the grid was blotted on both sides for 4 s and then vitrified by plunge freezing using Vitrobot Mark IV (FEI) into liquid ethane maintained at 93.0 K using an ethane cryostat (*46*). The Vitrobot chamber temperature was set to 37°C, and humidity was set to 100%. Micrograph movies were collected using a Titan Krios TEM (FEI) operating at 300 kV, with a K3 detector (Gatan Inc.). Pixel size was 1.08 Å, and dose per frame [32 after electron-event representation (EER) fractionation] was adjusted to 1.1 e^−^/Å^2^. A total of 2010 movies were collected in one session.

### *M. tuberculosis* FtsZ

*M. tuberculosis* FtsZ, purified and stored as above, was thawed on ice before being diluted to a final concentration of 0.2 mg/ml in ice-cold buffer HMK100 (*47*) [50 mM Hepes, 100 mM potassium acetate, 5 mM magnesium acetate, and 1 mM EGTA (pH 7.7)], and GMPCPP (Jena Bioscience) was added last to a final concentration of 0.5 mM. The sample was mixed by pipetting, incubated for 2 to 5 min at 20°C, and mixed again before 3 μl was applied to a glow-discharged (1 min at 40 mA) grid, either carbon on Cu/Rh (Quantifoil R2/2 200 mesh) or gold on gold UltrAuFoil R1.2/1.3 300 mesh. Without wait, the grid was blotted on both sides for 4 s and then vitrified by plunge freezing using Vitrobot Mark IV (FEI) into liquid ethane maintained at 93.0 K using an ethane cryostat (*46*). The Vitrobot chamber temperature was set to 10°C, and humidity was set to 100%. Movies with 0° stage tilt were collected using a Titan Krios TEM (FEI) operating at 300 kV, with a K3 detector (Gatan Inc.). Pixel size was 0.86 Å, dose per frame was adjusted to 1 e^−^/Å^2^, and 40 frames were recorded. A total of 13,020 movies were collected in two sessions. Movies with 40° stage tilt were collected using a Titan Krios TEM (FEI) operating at 300 kV, with a K2 detector (Gatan Inc.). Pixel size was 1.47 Å, dose per frame was adjusted to 1 e^−^/Å^2^, and 40 frames were recorded. Six hundred sixty movies were collected in one session.

### *P. dejongeii* BtubA*B

Mutant *P. dejongeii* BtubA*B protein purified and stored as above was thawed on ice before diluting to a final concentration of 0.21 mg/ml in ice-cold buffer HMK100 (*33*) [50 mM Hepes, 100 mM potassium acetate, 5 mM magnesium acetate, and 1 mM EGTA (pH 7.7)], with GMPCPP (Jena Bioscience) or GTP added last to final concentrations of 0.5 or 5 mM, respectively. Samples were mixed by pipetting, incubated at 20°C for 45 s (GMPCPP) or 2 min (GTP), and mixed again, and then 3 μl was applied to a freshly glow-discharged (1 min, 40 mA) gold-on-gold grid (UltrAuFoil R1.2/1.3 300 mesh). Without wait, the grid was blotted on both sides for 4 s and then vitrified by plunge freezing using Vitrobot Mark IV (FEI) into liquid ethane maintained at 93.0 K using an ethane cryostat (*46*). The Vitrobot chamber temperature was set to 10°C, and humidity was set to 100%. Movies were collected using a Titan Krios TEM (FEI) operating at 300 kV, with a K3 detector (Gatan Inc.). Pixel size was 1.1 Å, dose per frame was adjusted to 1.1 e^−^/Å^2^, and 40 frames were recorded. For the GMPCPP sample, a single grid was imaged. Four thousand seven hundred twenty-eight movies were collected with 0° stage tilt, and 1265 were collected with 40° stage tilt. For the GTP sample, 5311 movies were collected on a single grid with 0° stage tilt.

### Cryo-EM data processing

Unless stated otherwise, processing was with Relion 3.1 (*48*). CryoSPARC was version 3.1 (*49*).

### *D. melanogaster* αβ-tubulin heterodimers

#### GTP-bound sample

A schematic processing pipeline can be found in fig. S4. Two datasets were collected from a single grid and treated independently until the merging point indicated. Micrograph movies were imported and motion-corrected using the algorithm implemented within Relion. Contrast transfer function (CTF) estimation was performed using CTFFIND4 (*50*). Micrographs were filtered for high-resolution information content using Thon rings, and for ice thickness by calculating average pixel intensity in a central region, using the mrcfile.py library (*51*). Particles were picked using Relion’s Gaussian blob picker, before extraction in boxes of 110^2^ pixels at a nominal 1.72 Å/pixel. Several rounds of two-dimensional (2D) classifications were performed, using varying mask diameters and class numbers. Smaller masks were useful for gathering top views of the oblong tubulin heterodimer [for a similar approach, see (*52*)]. Initial 3D refinements used a 20-Å low pass–filtered tubulin heterodimer crystal structure (2Q1T). Particles were recentered/reextracted in boxes of 148^2^ pixels at a nominal 1.27 Å/pixel before 3D refinement and Bayesian polishing, in boxes of 148^2^ pixels using a calibrated pixel size of 1.244 Å/pixel. The best particles were recovered by 3D classification without alignment, with varying numbers of classes and values of the regularization parameter T. Datasets were merged for a final 3D refinement of 34,000 particles, yielding a 3.5-Å reconstruction.

#### GDP-bound sample

A schematic processing pipeline can be found in fig. S3. The overall approach was very similar to that used for the GDP-bound sample. Refinement of 69,000 high-quality particles yielded a final reconstruction at 3.2 Å.

### *D. melanogaster* MTs

Because we reconstructed “naked” MTs from 2D projection images, lacking any additional subunits indicating the positions of each of the α/β-tubulin heterodimers, we used an adaptation and extension of the method used by Lacey *et al.* (*53*) to be able to deal with the 26-fold pseudosymmetry of the 13 protofilament MTs imaged. Twofold pseudosymmetry arises because of α/β-tubulin being very similar in structure, and additional 13-fold pseudosymmetry arises because the 13 protofilaments are not equivalent because MTs are not truly helical and have a “seam,” where the B-lattice becomes an A-lattice.

All image processing was done in Relion 3.1 (*48*). On import, each EER exposure was dose-fractionated into 32 subframes that were then aligned against each other to yield motion-corrected images, using 8 and 5 tiles in X and Y. Subsequently, CTF parameters for each image were determined using CTFFIND 4.1 (*50*). One thousand seven hundred seventy images were retained after filtering out those images that produced poor CTF fits as determined by CTFFIND’s resolution estimation. One thousand two hundred twenty-two particles were picked manually and 2D-classified after extraction particle images in boxes of 270^2^ pixels and twofold binning (pixel size 2.16^2^ Å^2^). Six classes were used as the reference for automatic picking of helices as implemented in Relion, and 354,399 particles were picked. Extraction as before and subsequent 2D classification revealed some classes that were not 13 protofilament MTs, as obvious from by the lack of complete colinearity of the protofilaments with the MT axis. Only those classes showing fine details and also no twist of the protofilaments were retained, leading to a dataset of 223,884 particle images.

Using these particle images, a fully pseudo-symmetrized reconstruction was calculated with the helical parameters: twist −27.8° (~360°/13) and rise 9.51 Å (~43 Å*3/13). This averages all subunits onto all others, disregarding the seam and the differences between α- and β-tubulin. This and all subsequent 3D reconstructions used an overall mask approximately four dimers long and selecting the MT wall only. The resulting reconstruction at 4.4-Å resolution (after masked postprocessing in Relion) allowed the placing of five tubulin dimers (PDB 3J1T) in one randomly chosen protofilament. From the placed atomic model of a protofilament, a 20 Å–filtered map was generated in Chimera (*54*), which was used to create a mask that covers a single protofilament. At this point, particle images were reextracted in boxes of 400^2^ pixels and binned by 2/3 (box 266^2^ pixels, pixel size 1.624^2^ Å^2^). Using the Relion “--local_symmetry” option [see (*53*) for details], describing the 13-fold protofilament symmetry of MTs, and also overall helical symmetry, now following the heterodimers (twist = 0° and rise = 86.9 Å), another reconstruction was calculated (4.0-Å resolution after masked gold standard postprocessing in Relion) after particle polishing and 3D classifications, based on 44,105 particle images. The overall number of particle images was reduced intently at this step to enable the large symmetry expansion, which followed next.

Using the command “relion_particle_symmetry_expand --helix 1 --i run_data.star --o run_data_expanded26.star --twist 27.692307 --rise -10.0335231 --asu 26,” each particle image was added 25 times in all possible other positions within the 26-fold pseudo-redundant MT symmetry. In parallel, tubulin dimers from PDB 3J1T were fitted into the reconstructed map and only atoms from the loop filling the pocket in β-tubulin (residues 359 to 372) equivalent to the Taxol binding pocket in α-tubulin were left. This atomic model of the biggest difference between α- and β-tubulin was used to subtract map densities in all particle images (which were 26-fold symmetry expanded), which are not in or close to the Taxol binding pocket in α-tubulin or the equivalent pocket in β-tubulin. Several rounds of 3D classification without alignment were then run on these subtracted particle images, until the MT symmetry emerged in the best classes, which meant that only every second pocket along each protofilament was occupied by density (representing residues 359 to 372 in β-tubulin) and that the seam, where the pattern of alternating pockets changes, was clearly identifiable. Not allowing alignment was the most important setting here: No rotational/translational alignment of particles, only moving particles between classes, means that the particles from the symmetry expansion were simply sorted into the correct class that has the right positioning of the seam and along the protofilaments because the symmetry expansion provided all possible versions of each particle image in the dataset. Note that “–local_symmetry” was used in these classification runs because it substantially enhanced the signal by averaging correctly over all subunits. The final map was calculated from 39,594 particle images and was not using “gold standard” separation of half datasets, for purely technical reasons of Relion’s implementation. Therefore, the overall resolution was determined from the Fourier shell correlation (FSC) of the map against the final refined atomic model (see below).

### *M. tuberculosis* FtsZ

A schematic processing pipeline can be found in fig. S6. Datasets were treated independently until the merging point indicated. Motion correction of micrograph movies was performed using Relion’s own implementation, and motion-corrected micrographs were then imported into cryoSPARC. Patch-based CTF estimation was performed (needed for the tilted images), and a total of ~5 million filament segments were picked using Topaz 0.2.4 (*55*) via the cryoSPARC interface. Picks were extracted into 224^2^ pixel boxes, at 1.45 Å/pixel. 2D classification was used to remove bad particles, and 3.9 million particles remaining were merged and used for nonuniform refinement to give a low-resolution map. Particles were recentered and reextracted before another round of nonuniform refinement with little improvement. Further cleaning was carried out by reimporting the refinement results into Relion and performing 2D classification without alignment. A total of 1.9 million particles remaining were reimported to cryoSPARC for a final nonuniform refinement to give the final medium-resolution map.

### *P. dejongeii* BtubA*B

#### GMPCPP-bound sample

A schematic processing pipeline can be found in fig. S7. Tilted and untilted datasets were treated independently until the merging point indicated. Motion correction of micrograph movies was performed using Relion’s implementation, enabling Bayesian polishing at later steps. Per-patch CTF estimates, to account for sample tilt, were produced using Warp 1.0.9 (*56*). A total of 1.6 million filament segments (with Relion-compatible helical metadata) were picked at 45-Å intervals using Cryolo 1.7.4 in filament mode (*57*), before extraction in 440^2^ Å^2^ boxes, binned to 2.2 Å/pixel. Two rounds of 2D classifications were performed before recentering and reextracting the remaining 1 million particles, unbinned (1.1 Å/pixel). These particle images and associated metadata were imported into cryoSPARC using pyem (*58*). Homogeneous refinement yielded a map with a reported resolution of 2.9 Å; however, this was clearly a “mixed register” map—with both BtubA and BtubB subunits contributing to the density at all positions. The result of this mixed reconstruction (refined angles and shifts) was reimported into Relion. At this point, alternate picks along filaments were removed (possible because helical metadata was retained), as if the initial particle segments had been picked and extracted at 90-Å spacing. 3D classification without alignment was performed on the remaining 500,000 particles (using the angles and shifts from the cryoSPARC refinement) and was able to separate the two registers present in the consensus map. A total of 290,000 particles from classes corresponding to the two register possibilities were recentered and reextracted, with updated centers shifted along the *z* axis by half a monomer in either direction, before remerging—yielding a set of images, angles, and shifts corresponding to a reconstruction with a common register. These data were reimported to cryoSPARC for homogeneous refinement, yielding a 3.2-Å map with clearly distinguishable BtubA and BtubB subunits, indicating success of the approach. This reconstruction was again imported into Relion for Bayesian polishing, before reimporting to cryoSPARC for homogeneous refinement (2.7 Å) and, finally, nonuniform refinement (*59*) producing the final 2.6-Å reconstruction.

#### GTP-bound sample

A schematic processing pipeline can be found in fig. S9. Motion correction, CTF estimation, and helical picking were all carried out using the Relion implementations. A total of 750,000 particles were extracted in 440^2^ Å^2^ boxes, binned to 2.2 Å/pixel, before 2D classification. A total of 460,000 remaining particles were imported to cryoSPARC for homogeneous refinement to yield a low-resolution reconstruction (~8 Å).

### Model building and refinement

Data and model statistics are summarized in table S1.

### *D. melanogaster* αβ-tubulin heterodimers

#### GTP form 

For model building, a *D. melanogaster* α/β-tubulin heterodimer was homology-modeled using SWISSMODEL (*60*). The dimer was placed manually in the cryo-EM map and adjusted manually in MAIN (*61*), including the observed guanosine nucleotides. After manual building, the model was refined computationally using phenix.real_space_refine (*62*). After several cycles of manual building and real-space refinement, a satisfactory fit of the model to the map could be obtained and the model showed very good statistics: Molprobity score = 1.80, 85th percentile, with no Ramachandran outliers (*63*).

#### GDP form 

Model building and refinement proceeded as for the α/β-tubulin heterodimer in the GTP form. Molprobity score = 1.39, 97th percentile, with no Ramachandran outliers.

### *D. melanogaster* MT

Model building started with a PDB 3J1T αβ-tubulin heterodimer placed into a section of the final map, which was manually adjusted in MAIN (*61*) and refined computationally using phenix.real_space_refine (*62*). Once a satisfactory fit of the model to the map could be obtained, the heterodimer was repeatedly copied and placed in the map to describe three dimers in each of the 13 protofilaments. Last, the entire model was refined using phenix.read_space_refine against the entire MT map. The model versus map FSC was 0.5 at ~3.8-Å resolution, and the model showed excellent statistics: Molprobity score = 1.74, 88th percentile, with no Ramachandran outliers (*63*).

### *P. dejongeii* BtubA*B

For model building, a BtubAB heterodimer (PDB 2BTQ) was placed manually in the central section of the protofilament cryo-EM map and adjusted manually in MAIN (*61*), including the observed GMPCPP nucleotide. The resolution of the map made it possible and indeed straightforward to determine the register of the protofilament with respect to BtubA and BtubB subunits. After manual building, the model was refined computationally using phenix.real_space_refine (*62*). After several cycles of manual building and real-space refinement, a satisfactory fit of the model to the map could be obtained. Three BtubAB dimers were then placed in the protofilament cryo-EM map and refined computationally using phenix.real_space_refine against the entire map. The model showed very good statistics: Molprobity score = 2.03, 74th percentile, with no Ramachandran outliers.

### Structural analysis

All analyses were carried out using custom scripts written in R, making extensive use of the bio3d (*12*) and tidyverse (*64*) packages. Structure datasets were collected programmatically from the PDB (April 2021) by running individual phmmer (*65*) searches of PDB sequences with a representative from each of the subfamilies investigated.

Sequences from the downloaded PDB depositions were extracted and aligned as follows. First, a high-quality representative structure was selected for each subfamily, and family members were aligned for each superfamily via a hybrid approach combining structural and sequence alignments, including information from large alignments of homologs, using the PROMALS3D web server with defaults (*66*). Sequences were then aligned within each subfamily to the representative, using MUSCLE (*67*) (with some manual adjustments), before all sequences were combined into a super alignment on the basis of the representative alignment (with some manual adjustments). The resulting sequence alignments are in data file S3.

Structures were annotated by downloading the UniProt entry listed in the PDB annotation. Polymerization state was assigned semiautomatically on the basis of experimental technique but checked manually. Similarly, nucleotide state was assigned semiautomatically on the basis of ligand annotation in the PDB.

High-quality representatives of extant conformational states were selected by structurally aligning and clustering identically annotated (i.e., same sequence, same ligand, and same polymerization state) structures with RMSD of <1.0 Å, before choosing the highest-resolution example from the cluster. Sometimes, the cluster representative was manually adjusted to include a structure with a higher proportion of built residues, or rejected if other structure quality metrics were not very good.

Among the representatives, and using only the ungapped positions in the super alignment of representatives within each superfamily, the structurally conserved core was found using the bio3d::core.find routine implementing Gerstein’s algorithm (*12*, *68*, *69*). The structures were aligned on the core using bio3d::fit.xyz. Structure-based PCA was performed with bio3d::pca.pdbs.

## Supplementary Materials

### This PDF file includes:

Texts S1 and S2 Table S1 Figs. S1 to S12 Legends for movies S1 and S2 Legends for files S1 to S3
