# Supplementary material for: Diverse cytomotive actins and tubulins share a polymerization switch mechanism conferring robust dynamics
Source: Sci Adv. 2023 Mar 29;9(13):eadf3021. doi: 10.1126/sciadv.adf3021 (PMC10058229; doi:10.1126/sciadv.adf3021)
Supplement: Supplementary file 1 — Texts S1 and S2 Table S1 Figs. S1 to S12 Legends for movies S1 and S2 Legends for files S1 to S3 [file sciadv.adf3021_sm.pdf]

Science Advances
AAAS

Supplementary Materials for

Diverse cytomotive actins and tubulins share a polymerization switch mechanism conferring robust dynamics

James Mark Wagstaff *et al.*

Corresponding author: Jan Löwe, jyl@mrc-lmb.cam.ac.uk

Sci. Adv. **9**, eadf3021 (2023)
DOI: 10.1126/sciadv.adf3021

The PDF file includes:

Texts S1 and S2
Table S1
Figs. S1 to S12
Legends for movies S1 and S2
Legends for files S1 to S3

Other Supplementary Material for this manuscript includes the following:

Movies S1 and S2
Files S1 to S3

Supplementary Table

Supplementary Table S1. Cryo-EM data collection and model statistics.

**Sample**	Tubulin heterodimer<br>GDP	Tubulin heterodimer<br>GTP	Microtubule<br>GDP<br>13 protofilaments	BtubAB protofilament<br>GMPCPP<br>M-loop mutant:<br>A(R284G,K286D,<br>F287G)	FtsZ protofilament<br>GMPCPP
Organism	*D. melanogaster*	*D. melanogaster*	*D. melanogaster*	*P. dejongeii*	*M. tuberculosis*
EM Data Bank map ID	EMD-14147	EMD-14148	EMD-14150	EMD-14151	Not deposited
PDB model ID	PDB 7QUC	PDB 7QUD	PDB 7QUP	PDB 7QUQ	Not deposited
**Data collection and processing**					
Magnification	64,000	64,000	81,000	81,000	105,000/81,000
Voltage (kV)	300	300	300	300	300
Electron fluence ($e^-/Å^2$)	40	40	35	44	40
Defocus range (μm)	-1 to -3 (nominal)	-1 to -3 (nominal)	-1 to -3 (nominal)	-1 to -3 (nominal)	-1 to -3 (nominal)
Pixel size (Å)	1.244	1.244	1.080	1.080 (some 40° tilted)	0.86/1.47(40° tilted)
Symmetry imposed	C1	C1	local 13 pf MT	C1	C1
Initial particle images (no.)	6,500,000	1,695,000	354,399	1,600,000	5,000,000
Final particle images (no.)	68,851	34,175	39,594	290,000	1,900,000
Map resolution (Å)	3.2	3.5	Not gold standard	2.6 (anisotropic)	~7 (anisotropic)
FSC threshold	0.143	0.143	Not gold standard	0.143	0.143
**Model**					
Initial model used (PDB code)	SWISSMODEL	SWISSMODEL	3J1T	2BTQ	6YM1 / 3VOA
Model resolution (Å)	3.4	3.6	3.8	3.5	Rigid body only
FSC threshold	0.5	0.5	0.5	0.5	
Map sharpening B factor ($Å^2$)	-81	-61	-175	-33	
Model composition					
Non-hydrogen atoms	6,680	6,696	263,094	19,095	
Protein residues	842	844	33,189	2,463	
Nucleic acid residues	0	0	0	0	
Ligands	GTP: 1<br>GDP: 1<br>Mg: 1	GTP: 2<br>Mg: 1	GTP: 39<br>GDP: 39<br>Mg: 39	G2P (GMPCPP): 6	
R.M.S. deviations					
Bond lengths (Å)	0.004	0.004	0.005	0.003	
Bond angles (°)	0.557	0.610	0.744	0.754	
**Validation**					
MolProbity score	1.39	1.80	1.74	2.03	
Clashscore	7.16	11.47	9.15	15.88	
Poor rotamers (%)	0	2	2	3	
Ramachandran plot					
Favored (%)	98.2	96.5	96.2	95.3	
Allowed (%)	1.8	3.5	3.8	4.5	
Disallowed (%)	0	0	0	0	

Supplementary text

Supplementary Text S1. Notes on PCA.

Details of structural survey

PC values and annotations for all representative structures are contained in Supplementary Files S1 and S2: supp_table_pca_act.csv and supp_table_pca_tub.csv.

	**Actins**	**Tubulins**
Representatives used to search PDB	5AEY_C, 5JLF_D, 5LJV_D, 5MW1_D, 4CZJ_B, 4A2B_A, 6F95_B, 4XHN_A	1W59_A, 3CB2_A, 3ZID_A, 4EI7_A, 4XCQ_A, 3R4V_A, 2BTQ_A, 2BTQ_B, 3M89_A, 3VOA_A, 1RLU_A, 4B45_A, 5M8G_A, 5M8G_C
N of chains downloaded from PDB	459	1441
N of entries downloaded from PDB	233	329
N of chains used as cluster representatives for PCA	107	165
N of aligned, un-gapped "core" positions	143	205
N of invariant residues	18	17

Commentary on selected actin structures

In this section and the one for tubulins, outliers in the PC subspaces are briefly described, along with all structures whose polymerisation state was annotated "special/ambiguous" (coloured green in Figures 2e and 5c, marked here with an asterisk "*"), often also outliers.

Outliers

2ZWH_A – Model for the eukaryotic F-actin structure (Oda et al. 2009), derived from fibre diffraction data; structure performs poorly on validation metrics and was refined without modern tools.

5YU8_A – Cofilin(severing protein)-decorated eukaryotic actin filament (Tanaka et al. 2018), i.e. highly unstable. Filament structure, but conformation is closer to the monomer cluster.

1HLU_A – Interesting eukaryotic actin open monomer, but structure performs poorly on validation metrics and was refined without modern tools.

5WFN_A – Eukaryotic actin complex with leiomodin, a nucleator protein that binds at multiple sites on the monomer. Interestingly the conformation is *more* open than most monomers (the opposite of e.g. 4A62_B).

4A62_B* – ParM complex with fragment of ParR, its filament nucleator. The monomeric structure adopts a conformation similar to the polymerised protein.

Commentary on selected tubulin structures

Outliers

5H5I_A* – FtsZ R29A point mutant in which the energy barrier between the subunit switch states has been removed (Fujita et al. 2017).

6UMK_A, 6UNX_A, 6LL5_A – three representatives of the somewhat unusual FtsZs from *Klebsiella pneumoniae* and *Escherichia coli*.

6BBN_D – aβ-tubulin from a curved tubulin complex induced by the kinesin-13 Kif2A.

1W59_A*, 1W59_B* – two FtsZ chains, identical in sequence, form a pseudo-polymerised dimer inside a crystal.

6B0C_A*, 6B0C_B* – these two chains form a polymer of tubulin heterodimers around the outside of a microtubule, linked via kinesin-13s.

6V5V_G* – structure of gamma-tubulin in the native human gamma-tubulin ring complex.

7ANZ_A*, 7ANZ_B* – two gamma tubulins within a structure of gamma-Tubulin Small Complex (gTuSC).

Four unusual α-tubulin conformations, from polymer structures but which are found in the monomeric conformation cluster:

1TVK_A – electron crystallography structure.

6REV_A – Cryo-EM structure of human doublecortin (MT stabiliser) bound to 13-pf GDP-MT.

1JFF_A – electron crystallography structure of zinc-induced sheets.

6CVJ_A – Tau bound to MT by cryo-EM. Possibly a refinement mistake.

And the reverse, two α-tubulin monomer structures that lie close to the filament cluster.

6GWC_A – from a heterodimer in complex with a synthetic protein binding partner.

4FFB_A – from a heterodimer in complex with a TOG MT depolymerase domain.

Supplementary Text S2. Proteins used in this study.

Drosophila melanogaster tubulins

α1 tubulin at 84B (His$_6$-tag **[red]**, PC-tag **[cyan]**, tubulin M1 underlined):
M**HHHHHHEDQVDPRLIDGK**GGGGRPMRECISIHVGQAGVQIGNACWELYCLEHGIQPDGQMPSDKTVGGGDDSFNTFFSETGAGKHVPRAVFVDLEPTVVDEV
RTGTYRQLFHPEQLITGKEDAANNYARGHYTIGKEIVDLVLDRIRKLADQCTGLQGFLIFHSFGGGTGSGFTSLLMERLSVDYGKKSKLEFAIYPAPQVSTAV
VEPYNSILTTHTTLEHSDCAFMVDNEAIYDICRRNLDIERPTYTNLNRLIGQIVSSITASLRFDGALNVDLTEFQTNLVPYPRIHFPLVTYAPVISAEKAYHE
QLSVAEITNACFEPANQMVKCDPRHGKYMACCMLYRGDVVPKDVNAAIATIKTKRTIQFVDWCPTGFKVGINYQPPTVVPGGDLAKVQRAVCMLSNTTAIAEA
WARLDHKFDLMYAKRAFVHWYVGEGMEEGEFSEAREDLAALEKDYEEVGMDSGDGEGEGAEEY*

β1 tubulin at 56D isoform B (co-purified with tagged α1 tubulin 84B):
MREIVHIQAGQCGNQIGAKFWEIISDEHGIDATGAYHGDSDLQLERINVYYNEASGGKYVPRAVLVDLEPGTMDSVRSGPFGQIFRPDNFVFGQSGAGNNWAK
GHYTEGAELVDSVLDVVRKEAESCDCLQGFQLTHSLGGGTGSGMGTLLISKIREEYPDRIMNTYSVVPSPKVSDTVVEPYNATLSVHQLVENTDETYCIDNEA
LYDICFRTLKLTTPTYGDLNHLVSLTMSGVTTCLRFPGQLNADLRKLAVNMVPFPRLHFFMPGFAPLTSRGSQQYRALTVPELTQQMFDAKNMMAACDPRHGR
YLTVAAIFRGRMSMKEVDEQMLNIQNKNSSYFVEWIPNNVKTAVCDIPPRGLKMSATFIGNSTAIQELFKRISEQFTAMFRRKAFLHWYTGEGMDEMEFTEAE
SNMNDLVSEYQQYQEATADEDAEFEEEQEAEVDEN

Mycobacterium tuberculosis FtsZ

MtbFtsZ (His$_6$-tag and linker/PreScission cleavage site **[red]**, residues in **[cyan]** non-specific due to cloning strategy, FtsZ M1 underlined)
MSYYHHHHHHDYDIPTTLEVLFQ/G**PMG**TPPHNYLAVIKVVGIGGGGVNAVNRMIEQGLKGVEFIAINTDAQALLMSDADVKLDVGRDSTRGLGAGADPEVGR
KAAEDAKDEIEELLRGADMVFVTAGEGGGTGTGGAPVVASIARKLGALTVGVVTRPFSFEGKRRSNQAENGIAALRESCDTLIVIPNDRLLQMGDAAVSLMDA
FRSADEVLLNGVQGITDLITTPGLINVDFADVKGIMSGAGTALMGIGSARGEGRSLKAAEIAINSPLLEASMEGAQGVLMSIAGGSDLGLFEINEAASLVQDA
AHPDANIIFGTVIDDSLGDEVRVTVIAAGFDVSGPGRKPVMGETGGAHRIESAKAGKLTSTLFEPVDAVSVPLHTNGATLSIGGDDDDVDVPPFMRR*

Prosthecobacter dejongeii BtubA*B

BtubA*, (R284G, R286D, F287G, M-loop mutations underlined):
MKVNNTIVVSIGQAGNQIAASFWKTVCLEHGIDPLTGQTAPGVAPRGNWSSFFSKLGESSSGSYVPRAIMVDLEPSVIDNVKATSGSLFNPANLISRTEGAGG
NFAVGYLGAGREVLPEVMSRLDYEIDKCDNVGGIIVLHAIGGGTGSGFGALLIESLKEKYGEIPVLSCAVLPSPQVSSVVTEPYNTVFALNTLRRSADACLIF
DNEALFDLAHRKWNIESPTVDDLNLLITEALAGITASMRFSGFLTVEISLRELLTNLVPQPSLHFLMCAFAPLTPPDGSDGEELGIEEMIKSLFDNGSVFAAC
SPMEGRFLSTAVLYRGIMEDKPLADAALAAMREKLPLTYWIPTAFKIGYVEQPGISHRKSMVLLANNTEIARVLDRICHNFDKLWQRKAFANWYLNEGMSEEQ
INVLRASAQELVQSYQVAEESGAKAKVQDSAGDTGMRAAAAGVSDDARGSMSLRDLVDRRR

BtubB:
MREILSIHVGQCGNQIADSFWRLALREHGLTEAGTLKEGSNAAANSNMEVFFHKVRDGKYVPRAVLVDLEPGVIARIEGGDMSQLFDESSIVRKIPGAANNWA
RGYNVEGEKVIDQIMNVIDSAVEKTKGLQGFLMTHSIGGGSGSGLGSLILERLRQAYPKKRIFTFSVVPSPLISDSAVEPYNAILTLQRILDNADGAVLLDNE
ALFRIAKAKLNRSPNYMDLNNIIALIVSSVTASLRFPGKLNTDLSEFVTNLVPFPGNHFLTASFAPMRGAGQEGQVRTNFPDLARETFAQDNFTAAIDWQQGV
YLAASALFRGDVKAKDVDENMATIRKSLNYASYMPASGGLKLGYAETAPEGFASSGLALVNHTGIAAVFERLIAQFDIMFDNHAYTHWYENAGVSRDMMAKAR
NQIATLAQSYRDAS

Supplementary figures

Supplementary Figure S1. Actin PCA supplements

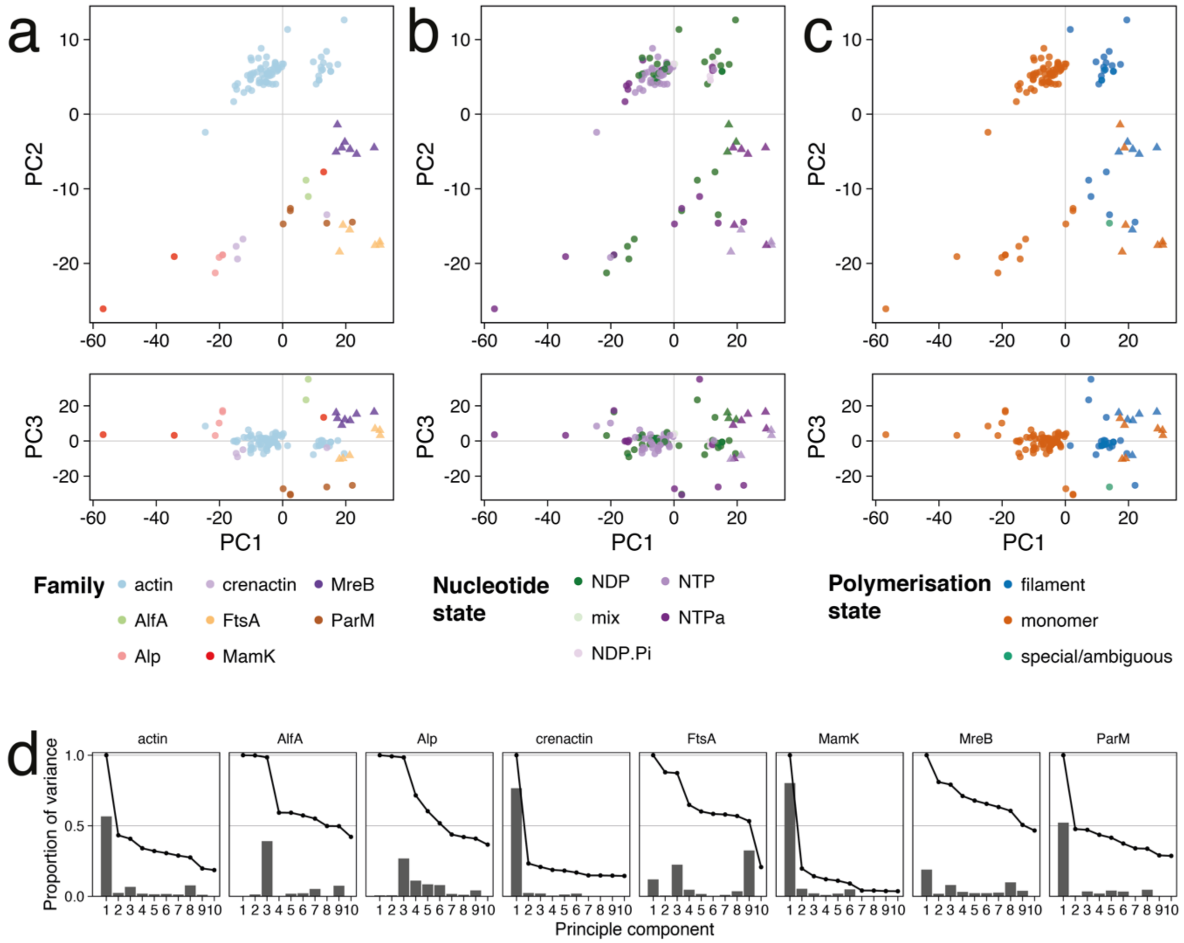

(a-c) Actin PC subspaces as in Figure 2, with the PC1-PC3 subspaces added below each plot. (d) Proportion of variance within each family's Cαs explained by each of the superfamily PCs. PC1 is descriptive for the (cytomotive) families with representatives of both polymerised and un-polymerised subunits. PC2 mostly describes differences between families so is not descriptive for any given family.

Supplementary Figure S2. Actin PCA with labelled structures

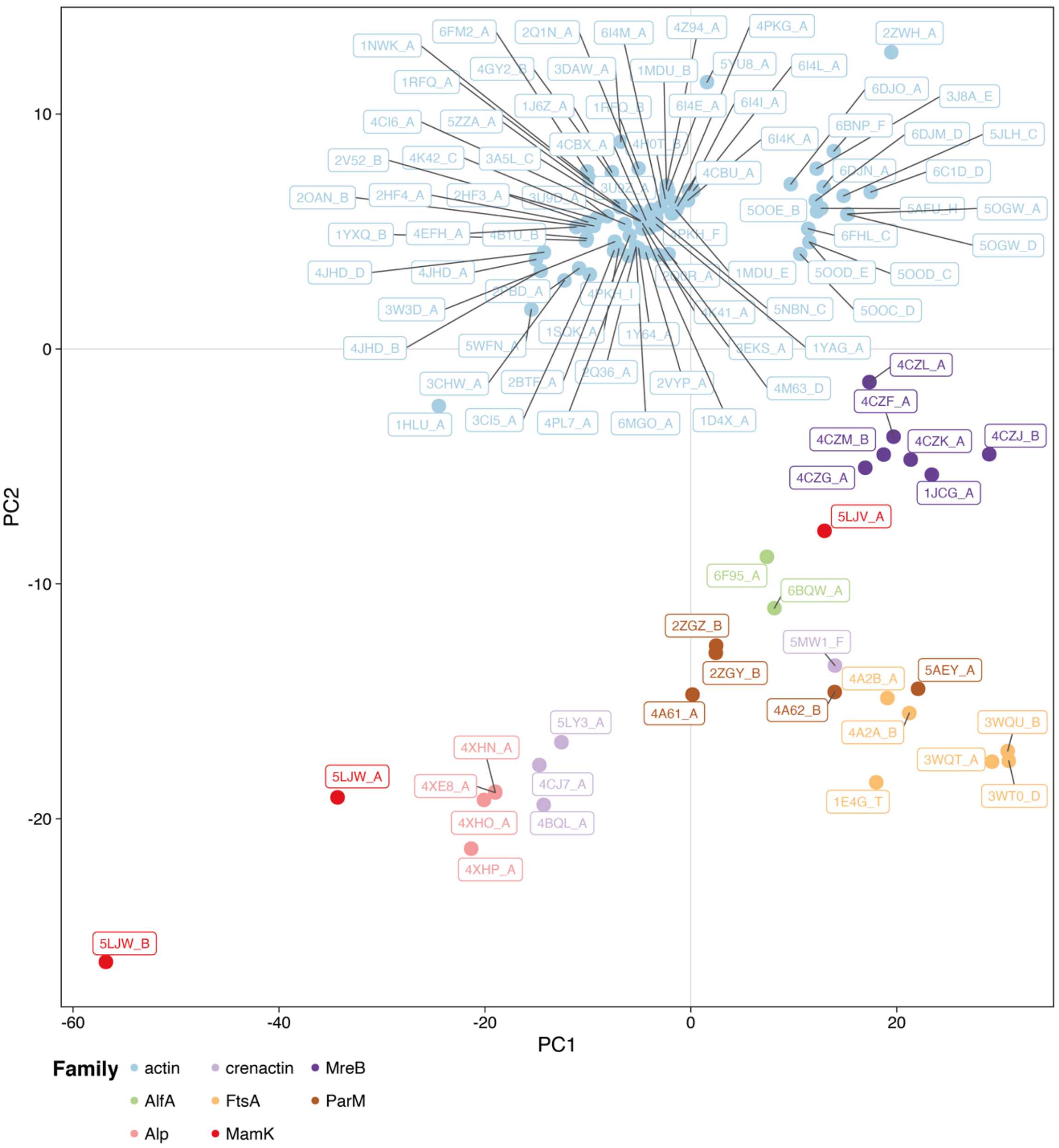

Actin PC1-PC2 subspace as in Figure 2c, with each representative structure labelled in the format '"PDB accession"_"chain id"'.

Supplementary Figure S3. *Drosophila melanogaster* (*Dm*) tubulin GDP cryo-EM processing scheme.

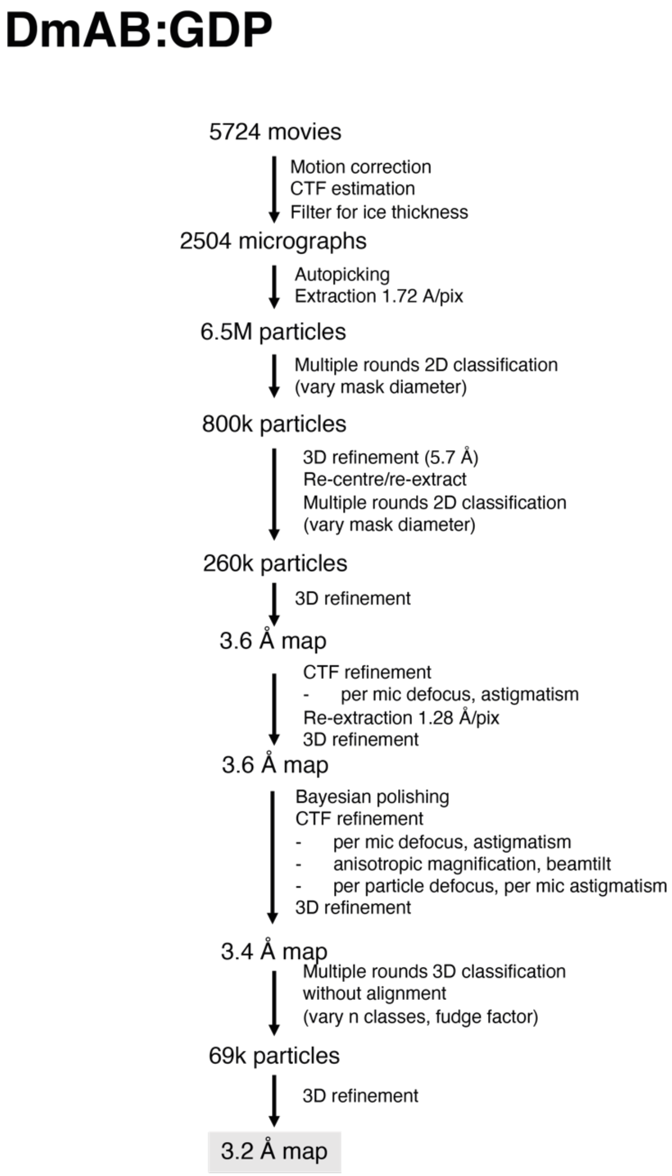

Supplementary Figure S4. *Drosophila melanogaster* (*Dm*) tubulin GTP cryo-EM processing scheme.

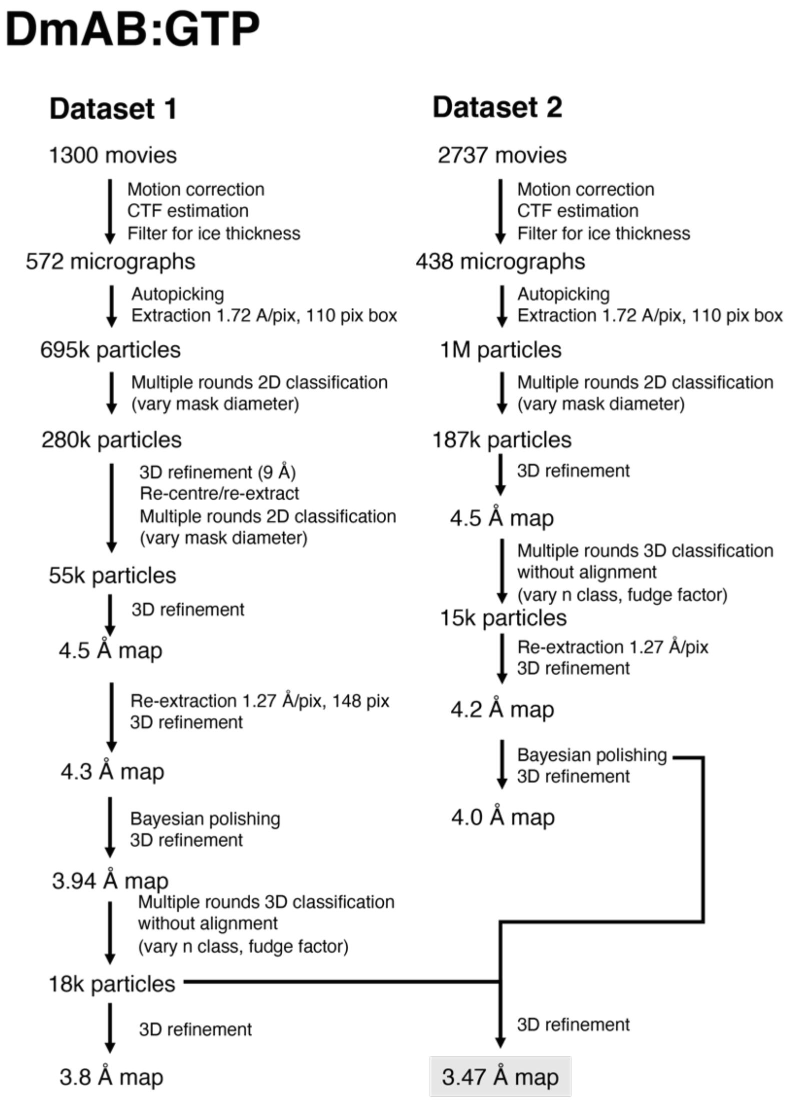

Supplementary Figure S5. Cryo-EM structure of *Mycobacterium tuberculosis* (*Mtb*) FtsZ, a single protofilament tubulin, reveals a strict assembly switch.

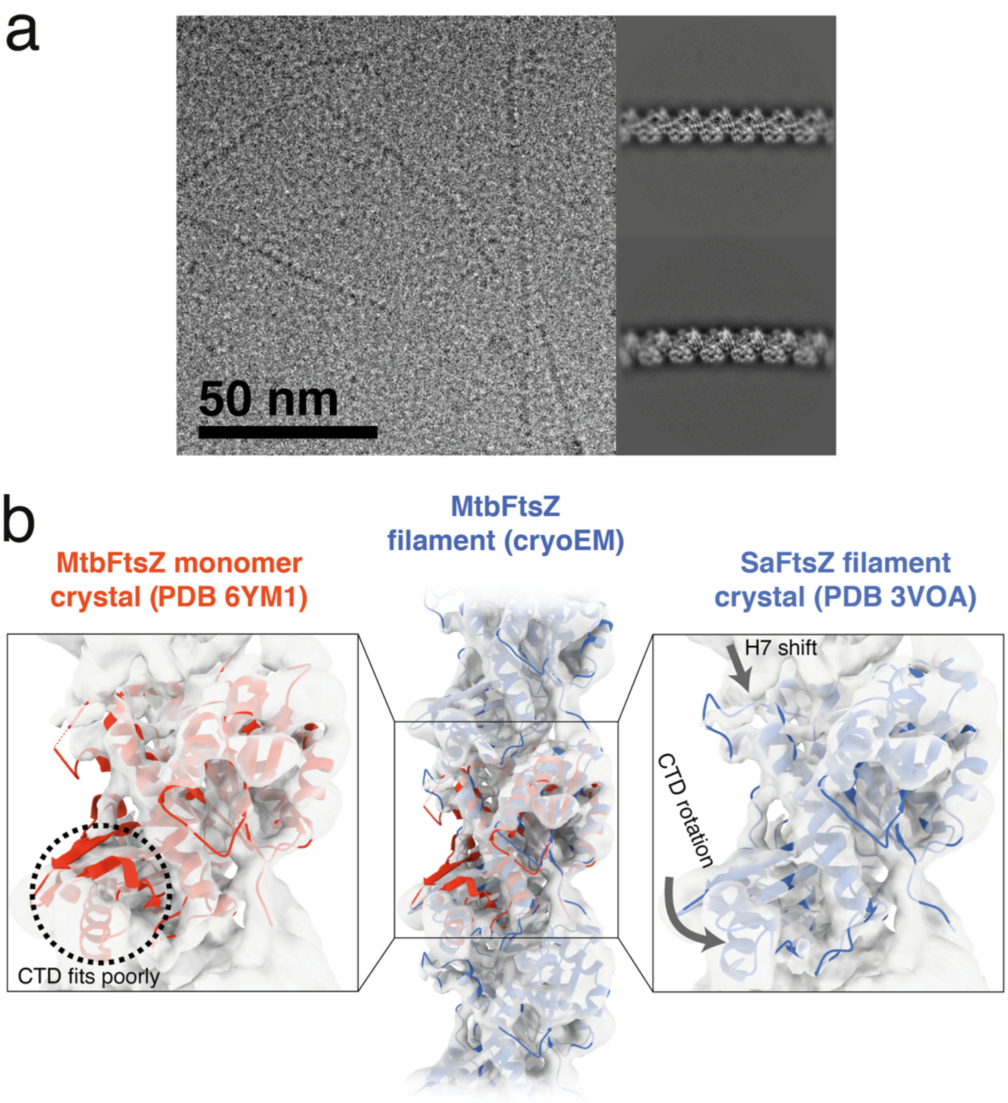

(a) Cryo-EM study of *Mycobacterium tuberculosis* (*Mtb*) FtsZ filaments. Representative micrograph and 2D class averages. Processing scheme can be found in Supplementary Figure S6. (b) Medium-resolution cryo-EM map of FtsZ filament from *M. tuberculosis*, reveals the assembly switch upon polymerisation. The map is compatible with the polymeric *S. aureus* FtsZ crystal structure (blue), but not the monomeric *M. tuberculosis* structure (orange). Map resolution is limited because of severe preferred orientation of the filaments after vitrification and the fact that the filaments are not helical (as was the case for *E. coli* FtsZ filaments, described in (J. M. Wagstaff et al. 2017))

Supplementary Figure S6. *Mycobacterium tuberculosis* (*Mtb*) FtsZ:GMPCPP cryo-EM processing scheme.

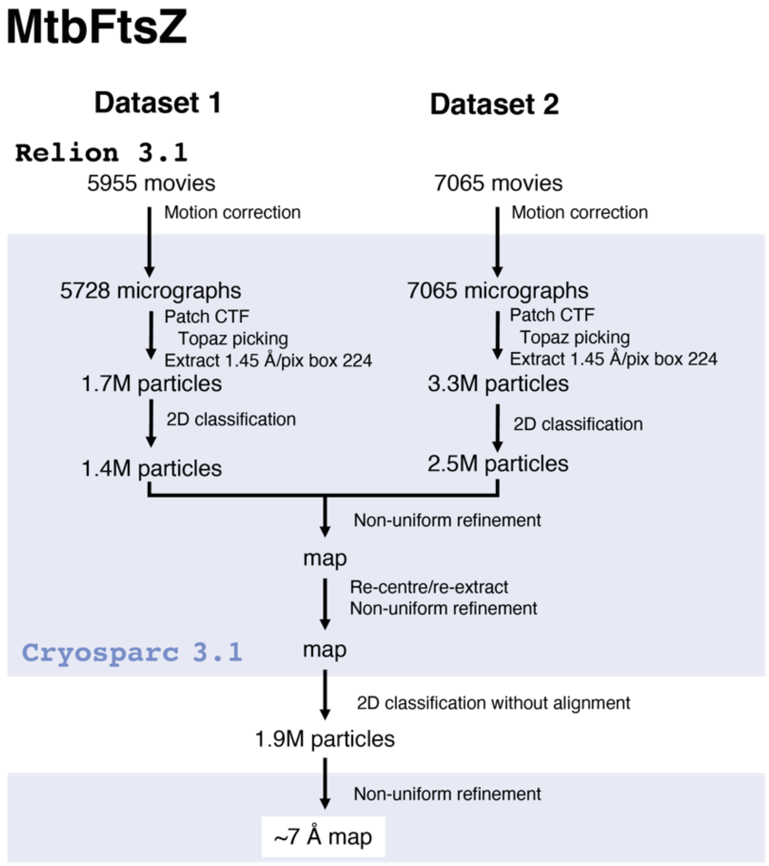

Supplementary Figure S7. BtubA*B:GMPCPP cryo-EM processing scheme.

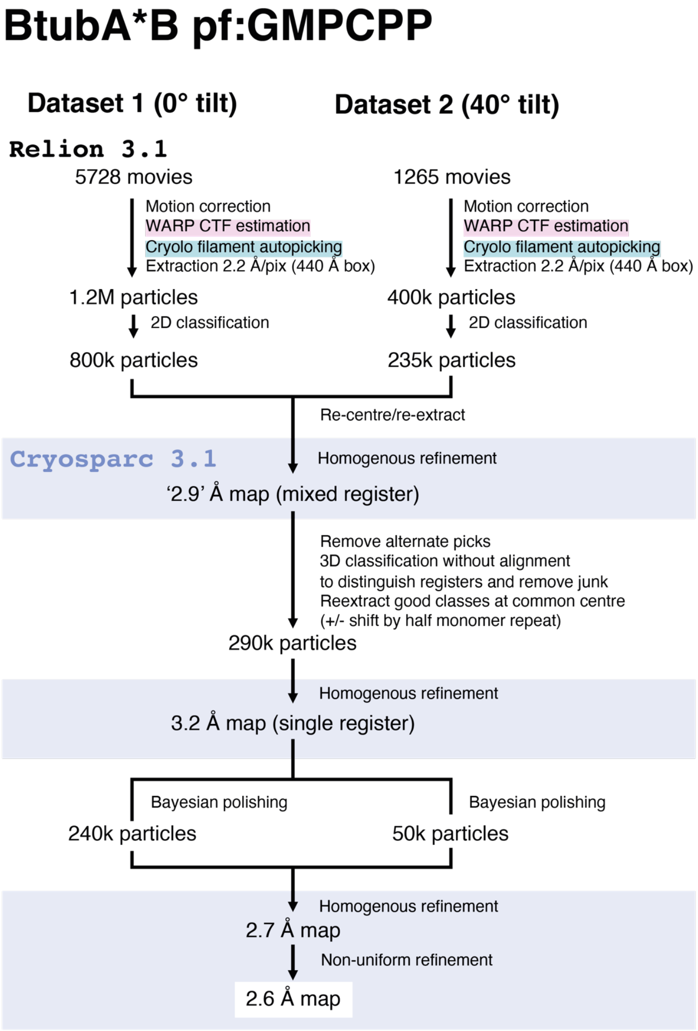

Supplementary Figure S8. BtubA*B polymerises with GTP to form single-stranded filaments.

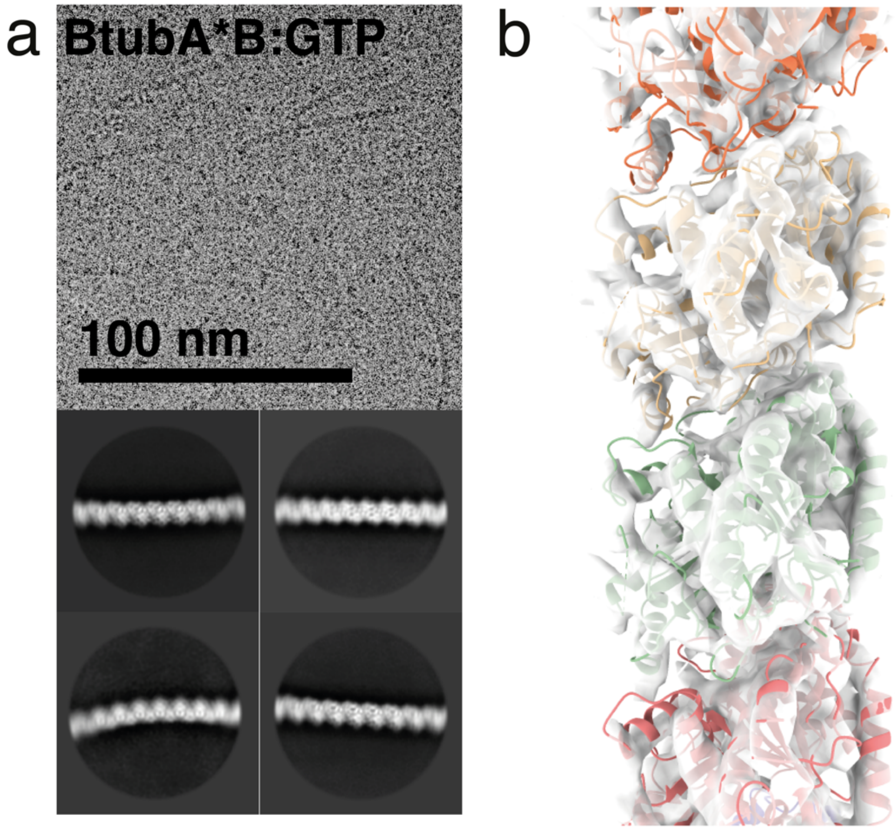

(a) Cryo-EM study of short and curved BtubAB filaments assembled with GTP. Representative micrograph and 2D class averages. (b) Medium resolution cryo-EM map (~ 8 Å resolution) of BtubA*B:GTP, with model of BtubAB filament rigid body fitted.

Supplementary Figure S9. BtubA*B:GTP cryo-EM processing scheme.

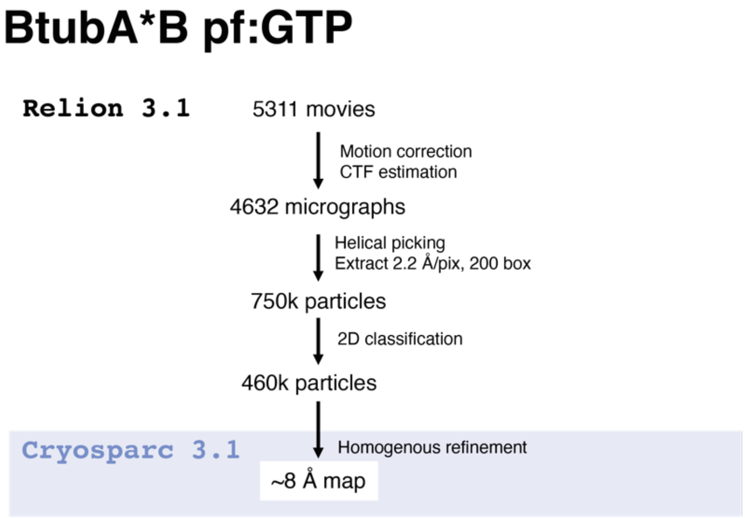

Supplementary Figure S10. Tubulin PCA supplements.

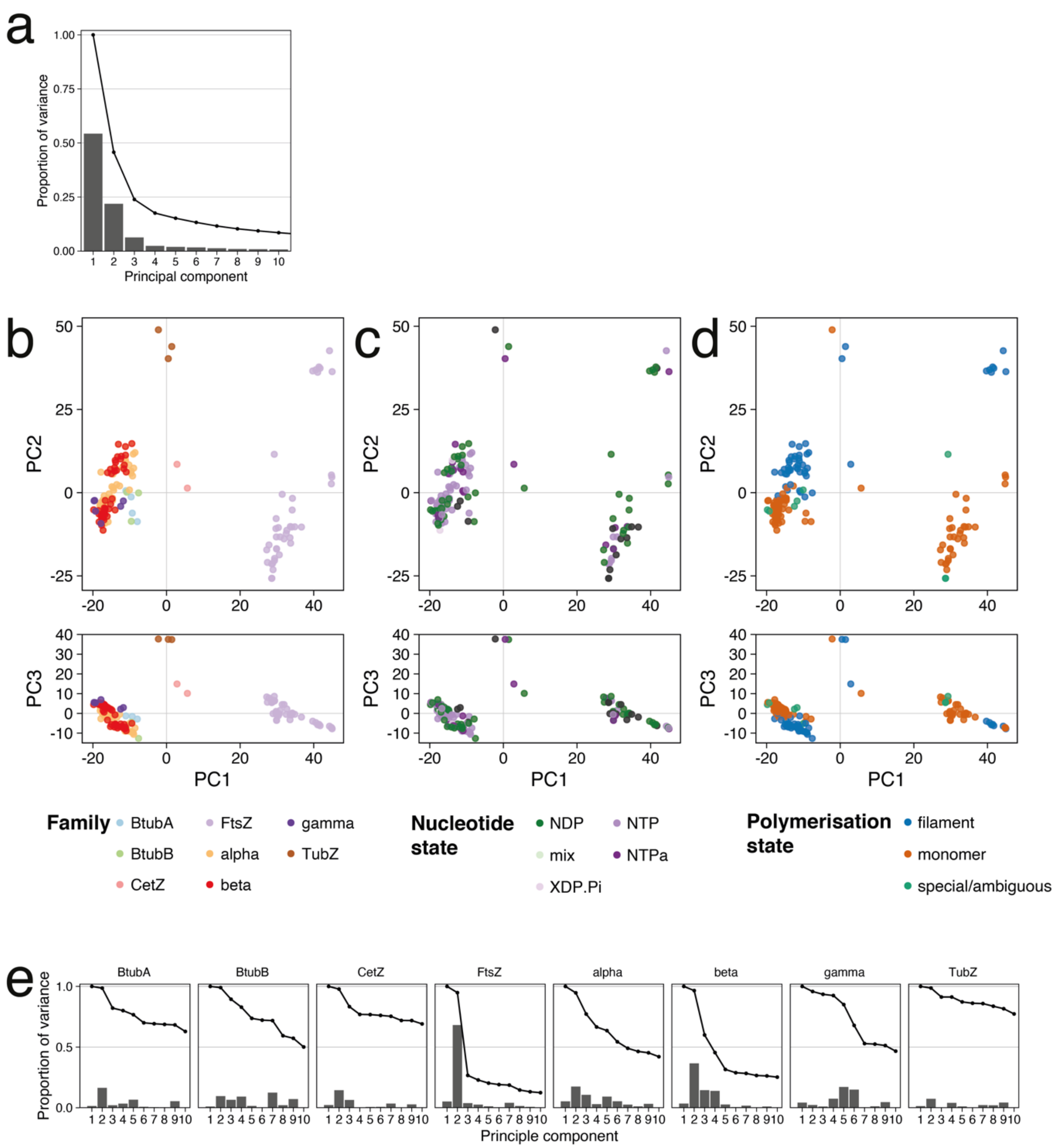

(a) Proportion of variance across all tubulin structures explained by the principal components (PC). (b-d) Tubulin PC subspaces as in Figure 5, with the PC1-PC3 subspaces added below each plot. (e) Proportion of variance within each family's Cαs explained by each of the superfamily PCs. PC2 is descriptive for the (cytomotive) families with representatives of both polymerised and un-polymerised subunits because it describes the polymerisation switch. PC1 mostly describes differences between families so is not descriptive for any given family.

Supplementary Figure S11. Tubulin PC1-PC2 subspace with labelled structures.

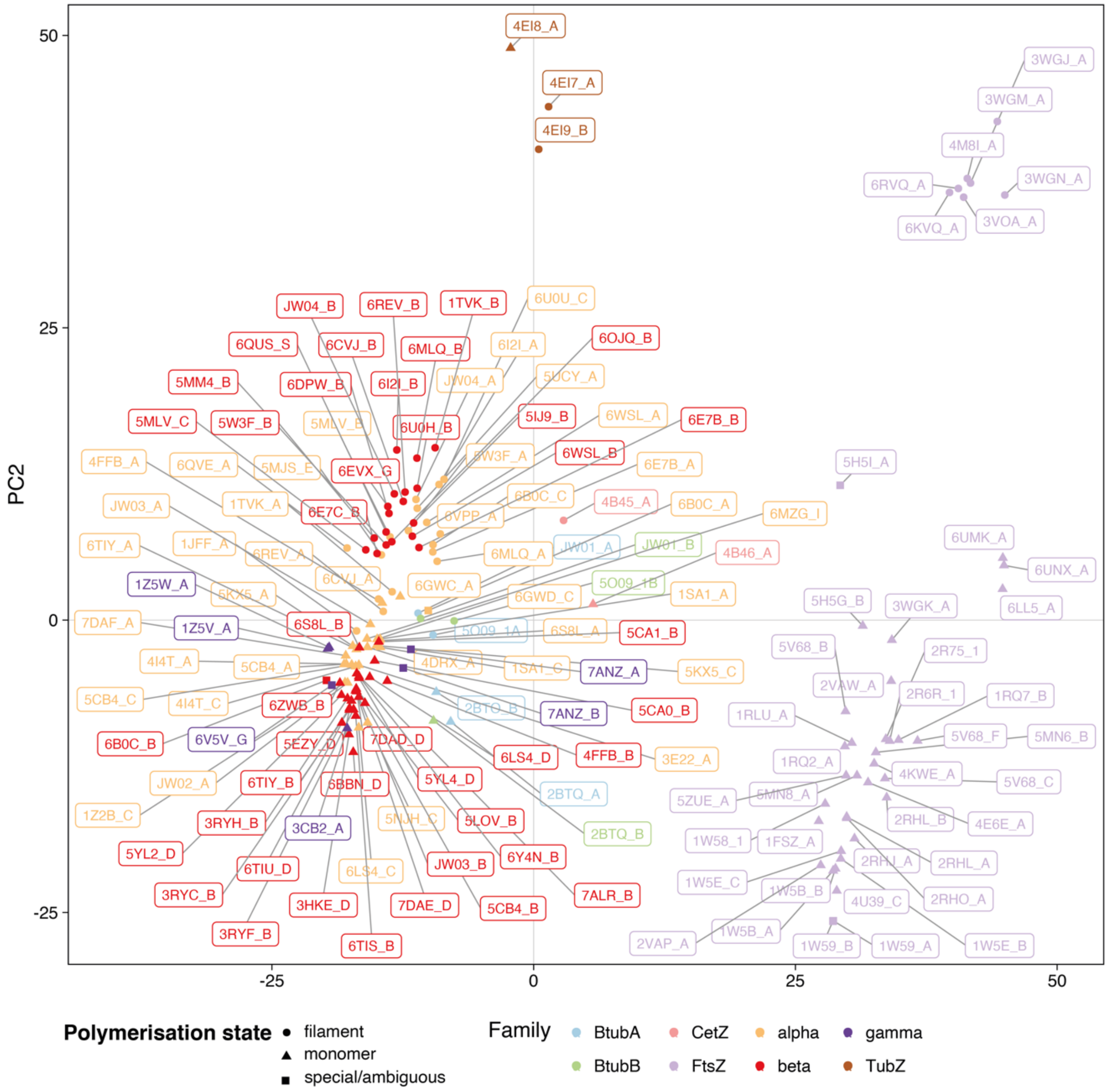

Tubulin PC1-PC2 subspace as in Figure 5a, with each representative structure labelled in the format '"PDB accession"_"chain id"'.

Supplementary Figure S12. Un-coupled kinetic and structural polarities in a filament without a cytomotive switch

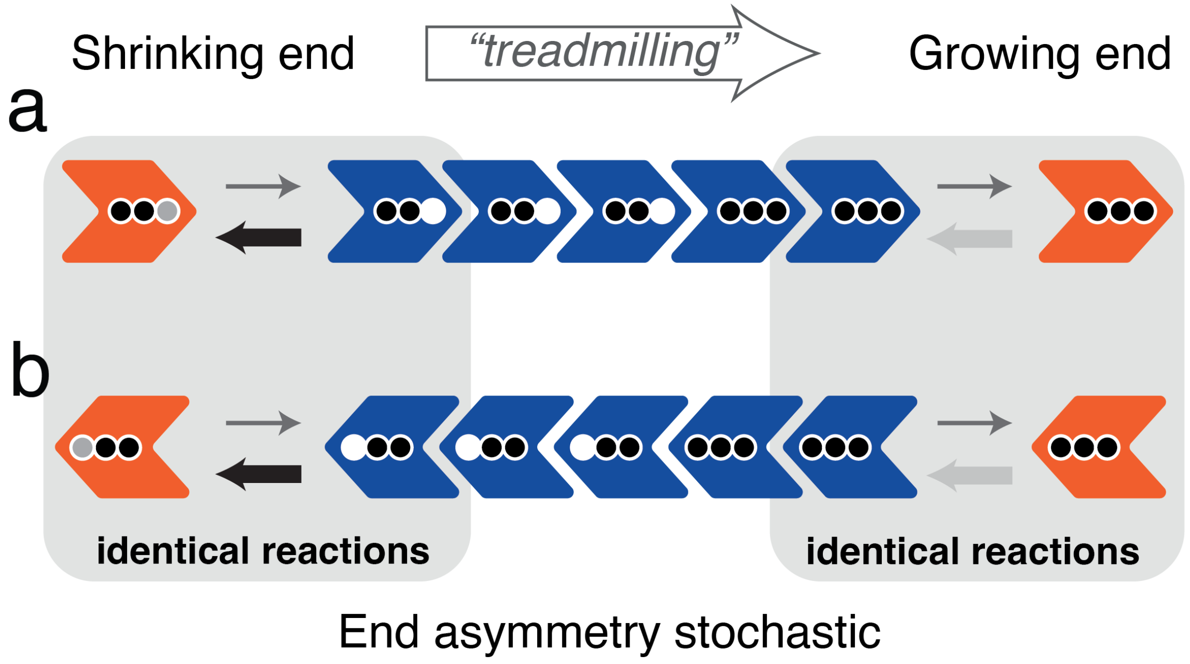

Two possible outcomes for dynamic polymerisation of one species of rigid subunits as in Fig 6a. These subunits can treadmill, but the direction in which they do so will be arbitrarily set by stochastically formed gradients of nucleotide hydrolysis within the filament. Thus, the kinetic (plus/minus) polarity will be uncoupled from the structural (pointed/notched end of chevrons) polarity. Such a filament will be less useful for many cytomotive tasks, as specific end binding is often used for positioning sub-cellular components.

Supplementary Movies

Supplementary Movie S1

Visualisation of principle components (PC) 1, 2 and 3, describing structural variations among actin superfamily members.

Supplementary Movie S2

Visualisation of principle components (PC) 1, 2 and 3, describing structural variations among tubulin superfamily members.

Supplementary Files

Supplementary File S1

Related to Supplementary Text S1: Notes on PCA. Principle component (PC) values and annotations for all representative actin-like structures are listed in the Microsoft Excel-readable comma separated values (CSV) file: supp_table_pca_act.csv.

Supplementary File S2

Related to Supplementary Text S1: Notes on PCA. Principle component (PC) values and annotations for all representative tubulin-like structures are listed in the Microsoft Excel-readable comma separated values (CSV) file: supp_table_pca_tub.csv.

Supplementary File S3

Sequence alignments for the PCA analyses of both actin and tubulin superfamilies. Zipped archive (.zip), containing readme.txt, which provides more information regarding the six files the archive contains. File: supp_seqs.zip.
